# Human galectin-9 potently enhances SARS-CoV-2 replication and inflammation in airway epithelial cells

**DOI:** 10.1093/jmcb/mjad030

**Published:** 2023-05-01

**Authors:** Li Du, Mohamed S Bouzidi, Akshay Gala, Fred Deiter, Jean-Noël Billaud, Stephen T Yeung, Prerna Dabral, Jing Jin, Graham Simmons, Zain Y Dossani, Toshiro Niki, Lishomwa C Ndhlovu, John R Greenland, Satish K Pillai

**Affiliations:** Vitalant Research Institute, San Francisco, CA 94105, USA; Department of Laboratory Medicine, University of California, San Francisco, CA 94143-0134, USA; Vitalant Research Institute, San Francisco, CA 94105, USA; Department of Laboratory Medicine, University of California, San Francisco, CA 94143-0134, USA; Vitalant Research Institute, San Francisco, CA 94105, USA; Department of Laboratory Medicine, University of California, San Francisco, CA 94143-0134, USA; Department of Medicine, University of California, San Francisco, CA 94143-0410, USA; Veterans Affairs Health Care System, San Francisco, CA 94121, USA; QIAGEN Digital Insights, Redwood City, CA 94063, USA; Division of Infectious Diseases, Department of Medicine, Weill Cornell Medicine, New York, NY 10021, USA; Vitalant Research Institute, San Francisco, CA 94105, USA; Department of Laboratory Medicine, University of California, San Francisco, CA 94143-0134, USA; Vitalant Research Institute, San Francisco, CA 94105, USA; Department of Laboratory Medicine, University of California, San Francisco, CA 94143-0134, USA; Vitalant Research Institute, San Francisco, CA 94105, USA; Department of Laboratory Medicine, University of California, San Francisco, CA 94143-0134, USA; Vitalant Research Institute, San Francisco, CA 94105, USA; Department of Laboratory Medicine, University of California, San Francisco, CA 94143-0134, USA; Department of Immunology, Kagawa University, Kagawa 760-0016, Japan; Division of Infectious Diseases, Department of Medicine, Weill Cornell Medicine, New York, NY 10021, USA; Department of Medicine, University of California, San Francisco, CA 94143-0410, USA; Veterans Affairs Health Care System, San Francisco, CA 94121, USA; Vitalant Research Institute, San Francisco, CA 94105, USA; Department of Laboratory Medicine, University of California, San Francisco, CA 94143-0134, USA

**Keywords:** SARS-CoV-2, galectin-9, inflammation, airway epithelial cells

## Abstract

The severe acute respiratory syndrome coronavirus 2 (SARS-CoV-2) pandemic has caused a global economic and health crisis. Recently, plasma levels of galectin-9 (Gal-9), a β-galactoside-binding lectin involved in immune regulation and viral immunopathogenesis, were reported to be elevated in the setting of severe COVID-19 disease. However, the impact of Gal-9 on SARS-CoV-2 infection and immunopathology remained to be elucidated. In this study, we demonstrate that Gal-9 treatment potently enhances SARS-CoV-2 replication in human airway epithelial cells (AECs), including immortalized AECs and primary AECs cultured at the air–liquid interface. Gal-9–glycan interactions promote SARS-CoV-2 attachment and entry into AECs in an angiotensin-converting enzyme 2 (ACE2)-dependent manner, enhancing the binding of the viral spike protein to ACE2. Transcriptomic analysis revealed that Gal-9 and SARS-CoV-2 infection synergistically induced the expression of key pro-inflammatory programs in AECs, including the IL-6, IL-8, IL-17, EIF2, and TNFα signaling pathways. Our findings suggest that manipulation of Gal-9 should be explored as a therapeutic strategy for SARS-CoV-2 infection.

## Introduction

The first known case of coronavirus disease 2019 (COVID-19), caused by severe acute respiratory syndrome coronavirus 2 (SARS-CoV-2) infection, was reported in December 2019. Rapidly, COVID-19 cases were reported worldwide. To date, SARS-CoV-2 has accounted for >620 million infections and >6.5 million deaths worldwide (Dong et al., 2020). The rapid spread of SARS-CoV-2 continues to have a major impact on global health and the economy. COVID-19 is mainly characterized by pneumonia, including fever, cough, and chest discomfort, and in severe cases, dyspnea and lung infiltration (Hu et al., 2021).

The major cause of death in COVID-19 cases is acute respiratory distress syndrome accompanied by a cytokine storm (Hojyo et al., 2020). Several reports have identified specific circulating proteins and cytokines in blood plasma that are elevated in the setting of COVID-19 and may constitute clinically useful disease biomarkers (Del Valle et al., 2020; Han et al., 2020). One such specific protein is galectin-9 (Gal-9). Recent studies have revealed that plasma Gal-9 levels are elevated in COVID-19 patients and are positively correlated with COVID-19 severity (Bai et al., 2021; Bozorgmehr et al., 2021; Chen et al., 2021; Patel et al., 2021). Furthermore, plasma levels of Gal-9 during COVID-19 were positively correlated with that of key pro-inflammatory cytokines, including interleukin 6 (IL-6), interferon gamma-induced protein 10, and tumor necrosis factor alpha (TNFα; Bozorgmehr et al., 2021). However, the mechanism linking Gal-9 to severe COVID-19 disease remains to be elucidated.

Gal-9, encoded by the *LGALS9* gene, belongs to the galectin family, which includes 15 carbohydrate-binding proteins sharing a common carbohydrate-recognition domain (CRD; Miyanishi et al., 2007). Conserved CRDs can bind to β-galactoside-containing glycans (Miyanishi et al., 2007). Many members of the galectin family, including Gal-9, are able to recognize poly-N-acetyllactosamine (poly-LacNAc) structures occurring in both N- and O-linked glycans (Lujan et al., 2018). Gal-9 is known for its regulation of immune responses and viral pathogenesis through glycan-mediated recognition. It is ubiquitously expressed in different tissues and cells in humans (e.g. endothelial cells, T lymphocytes, dendritic cells, macrophages, and intestinal epithelial cells) and is localized in the extracellular matrix, surface, cytoplasm, and nucleus of cells (Shahbaz et al., 2020). Circulating levels of Gal-9 serve as sensitive and non-invasive biomarkers in a broad range of conditions, including cancer, autoimmunity, and infectious diseases, and the roles of Gal-9 vary with respect to cell type and disease state (Moar and Tandon, 2021). For example, Gal-9 suppresses antigen-specific CD8^+^ T cell effector functions via the interaction with its receptor, TIM-3 (Sehrawat et al., 2010), and the Gal-9/TIM-3 axis promotes tumor survival through the cross-talk with the PD-1 immune checkpoint (Yang et al., 2021). The functions of Gal-9 in the inflammatory response have been studied extensively. Gal-9 enhances cytokine secretion in the human mast cell line (Kojima et al., 2014) and potentiates the secretion of pro-inflammatory cytokines in inflammatory models of arthritis via the interaction with TIM-3 (Anderson et al., 2007). In specific relevance to viral pathogenesis, Gal-9 can bind to glycan structures expressed on the surface of both host cells and microorganisms to modulate antiviral immunity and to promote or inhibit viral infection and replication (Machala et al., 2019). Gal-9 has been demonstrated to inhibit human cytomegalovirus infection by its CRDs (Machala et al., 2019). In hepatitis C virus (HCV)-infected individuals, virus infection induces Gal-9 secretion, which in turn induces pro-inflammatory cytokines leading to depletion of CD4^+^ T cells, apoptosis of HCV-specific cytotoxic T cells, and expansion of regulatory T cells (Zhuo et al., 2017). Gal-9 is elevated in human immunodeficiency virus-1 (HIV-1)-infected individuals (Abdel-Mohsen et al., 2016; Premeaux et al., 2019), mediates HIV-1 transcription and reactivation (Abdel-Mohsen et al., 2016; Sanz et al., 2021), and potentiates HIV-1 infection by regulating the T cell surface redox environment (Bi et al., 2011). These previous reports provide compelling evidence of the diverse roles of Gal-9 in viral infection and virus-associated immunopathology.

To date, a causal role of Gal-9 in SARS-CoV-2 pathology has not been demonstrated. To address this gap, we investigated the effects of Gal-9 treatment on SARS-CoV-2 replication and pro-inflammatory signaling in immortalized and primary human airway epithelial cells (AECs). Our data show that Gal-9 facilitates SARS-CoV-2 replication and promotes virus-associated immunopathology in the human airway, motivating exploration into Gal-9 manipulation as a therapeutic strategy for COVID-19 disease.

## Results

### Gal-9 potently enhances SARS-CoV-2 replication in Calu-3 cells

**Figure 1:**
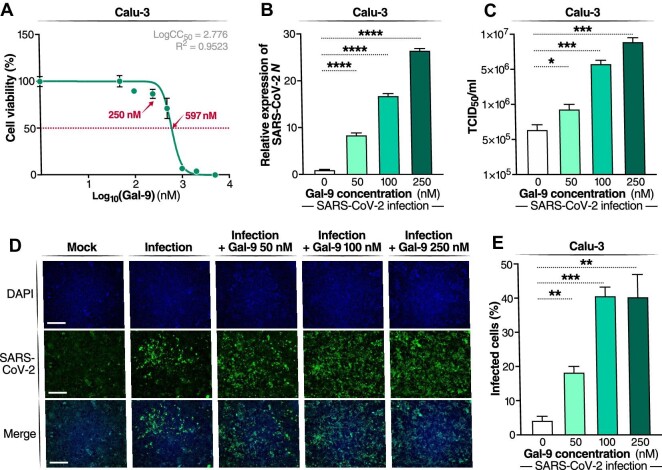
Gal-9 increases virus production in SARS-CoV-2-infected Calu-3 cells. (**A**) Cellular toxicity was examined in Calu-3 cells using the MTT assay and expressed as relative cell viability compared to Gal-9-untreated control (set at 100%). The logCC_50_ value for Gal-9 is displayed. The red arrows represent 250 nM and the CC_50_ value (597 nM) of Gal-9, respectively. (**B**–**E**) Calu-3 cells were pre-treated with Gal-9 at the indicated concentrations for 6 h, followed by infection with SARS-CoV-2 (MOI = 0.01) for 24 h in the presence of Gal-9. (**B**) Cells were then collected for RNA isolation. The effect of Gal-9 on viral *N* gene expression was measured by RT–qPCR. (**C**) Infectious virus release in the supernatant was measured using TCID_50_. (**D**) Immunofluorescence staining of these cells with DAPI (blue) and anti-N antibody (green). Scale bar, 500 μm. (**E**) Percentage of SARS-CoV-2-infected cells in total Calu-3 cells. Data are representative of the results of three independent experiments (mean ± SEM). Statistical significance was analyzed by the *t*-test. *P ≤ 0.05; **P ≤ 0.01; ***P ≤ 0.001; ****P ≤ 0.0001.

To investigate the impact of Gal-9 on SARS-CoV-2 infection, we first determined the natural expression of Gal-9 in human AECs with or without SARS-CoV-2 infection. In line with the undetected Gal-9 protein expression in alveolar epithelial cells (Lindskog, 2016), the mean Gal-9 level in the culture medium or on the cell surface of Calu-3 cells and primary AECs was negligible (Supplementary Figure S1). Moreover, the Gal-9 level was unaffected by SARS-CoV-2 infection (Supplementary Figure S1), implying that the elevated plasma Gal-9 level observed in COVID-19 patients is derived from other cell sources. Therefore, we used a stable form of recombinant Gal-9 in human AECs to mimic endogenously produced Gal-9. To optimize dosing for *in vitro* experiments, we relied on previously published Gal-9 concentrations in COVID-19 patients and the determined cytotoxicity of the recombinant Gal-9. The mean plasma Gal-9 concentration in COVID-19 patients was 2250 ng/ml, compared to 450 ng/ml in healthy controls (Iwasaki-Hozumi et al., 2021), and Gal-9 expression is significantly higher in pulmonary tissues than in plasma (Matsumoto et al., 2013). The 50% cytotoxic concentration (CC_50_) value for Gal-9 in Calu-3 cells was 597 nM (19700 ng/ml) as determined by the MTT assay (Figure 1A). Based on these criteria, we chose to test the effects of Gal-9 on SARS-CoV-2 replication at 50 nM (1650 ng/ml), 100 nM (3295 ng/ml), and 250 nM (8235 ng/ml) concentrations. Calu-3 cells were pre-treated with Gal-9 for 6 h before viral infection (MOI = 0.01), and Gal-9 was maintained in the medium until 24 h following infection. SARS-CoV-2 infection, as measured by the quantitation of viral nucleocapsid (*N*) gene expression, was increased significantly by treatment with Gal-9 in a dose-dependent manner (*P *< 0.0001), with up to 27-fold induction at the highest concentration of Gal-9 (Figure 1B). Similarly, the release of infectious virus in the supernatant was enhanced significantly by Gal-9 in a dose-dependent manner (*P* < 0.05), as measured by the median tissue culture infectious dose (TCID_50_) (Figure 1C). The enhancement of virus production by Gal-9 was confirmed by specific staining of viral N protein using the immunofluorescence assay (Figure 1D and E). Taken together, these data demonstrate that Gal-9 increases SARS-CoV-2 viral production in susceptible Calu-3 cells.

### Gal-9 enhances SARS-CoV-2 entry in an ACE2-dependent manner

To investigate the stage of the virus replication cycle impacted by Gal-9, we treated Calu-3 cells with Gal-9 at a concentration of 250 nM, chosen based on our toxicity and dose–response analyses. Calu-3 cells were treated with Gal-9 before or after virus infection. The protocols are illustrated in Supplementary Figure S2A. Gal-9-mediated enhancement of virus production was significantly higher in cells with pre-infection Gal-9 treatment than in cells with post-infection treatment (*P *< 0.05) (Supplementary Figure S2B). These results suggest that Gal-9 likely impacts the early stage of the SARS-CoV-2 viral life cycle.

**Figure 2:**
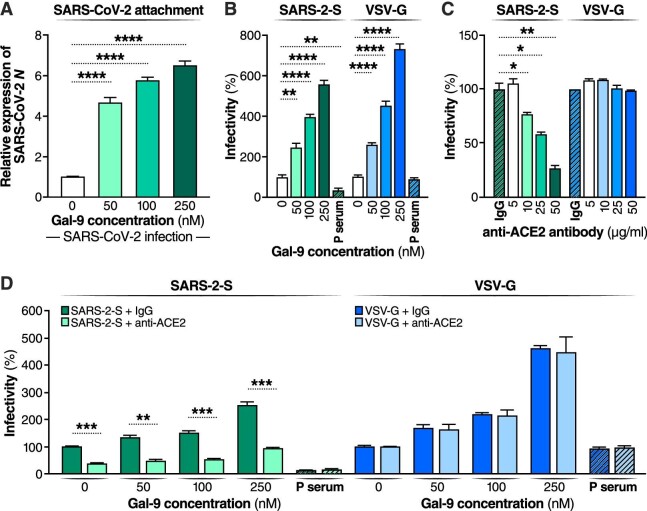
Gal-9 facilitates the cell-surface attachment and entry of SARS-CoV-2. (**A**) Attached SARS-CoV-2 virions on the cell surface were detected by RT–qPCR. Calu-3 cells were pre-treated with Gal-9 for 1 h and then incubated with SARS-CoV-2 (MOI = 0.01) in solutions with or without Gal-9 at the indicated concentrations at 4°C for 2 h. Cells were washed three times with PBS and harvested for RNA isolation and RT–qPCR measurement of SARS-CoV-2 *N* gene expression. (**B**) Calu-3 cells were exposed to Gal-9 for 6 h and then infected with SARS-2-S pseudotyped virus or VSV-G pseudotyped virus in solutions containing Gal-9 at the indicated concentrations. Pseudotyped viral entry was analyzed by luciferase activity at 24 hpi. Positive serum predetermined to possess anti-SARS-CoV-2 neutralizing activity was used as a negative control. Luciferase signals obtained in the absence of Gal-9 were used for normalization. (**C**) Relative infectivity of SARS-2-S pseudotyped virus and VSV-G pseudotyped virus in Calu-3 cells treated with anti-ACE2 antibody at the indicated concentrations. (**D**) Calu-3 cells were pre-treated with Gal-9 for 6 h and then pre-incubated with anti-ACE2 or control antibody (25 μg/ml) for 1 h, and cells were inoculated with SARS-2-S pseudotyped or VSV-G pseudotyped virus in solutions containing Gal-9 at the indicated concentrations. At 24 hpi, pseudotyped viral entry was analyzed by luciferase activity. Luciferase signals obtained in the absence of both Gal-9 and anti-ACE2 antibodies were used for normalization. Data are representative of the results of three independent experiments (mean ± SEM). Statistical significance was analyzed by the *t*-test. *P ≤ 0.05; **P ≤ 0.01; ***P ≤ 0.001; ****P ≤ 0.0001.

To determine whether Gal-9 affects SARS-CoV-2 viral entry, we first examined the cell-surface attachment of SARS-CoV-2. Cells were incubated with SARS-CoV-2 at 4°C for 2 h, and attached SARS-CoV-2 viral particles were detected after washing the cells three times. Gal-9 induced a substantial, highly significant (*P* < 0.0001), and dose-dependent increase in SARS-CoV-2 cell-surface attachment (Figure 2A). We next determined the capacity of Gal-9 to affect the entry of VSV-SARS-CoV-2 spike-ΔG-luciferase reporter pseudovirus (hereafter referred to as SARS-2-S) into Calu-3 cells. Positive serum (P serum), which was predetermined to possess SARS-CoV-2 neutralizing activity, potently reduced SARS-2-S infection (*P* < 0.01) but did not suppress the infection of VSV-spike G glycoprotein-luciferase reporter pseudovirus (hereafter referred to as VSV-G) (Figure 2B). Gal-9 markedly enhanced SARS-2-S infection in a dose-dependent manner in Calu-3 cells that endogenously express angiotensin-converting enzyme 2 (ACE2) and TMPRSS2 (*P* < 0.01) (Figure 2B), indicating that Gal-9 can potentiate SARS-CoV-2 attachment and entry. Unexpectedly, the entry of VSV-G was also significantly enhanced by Gal-9 treatment (*P* < 0.0001), suggesting that this pro-viral activity may be generalizable to other viral taxa.

ACE2 has been identified as the critical receptor for SARS-CoV-2 binding and entry (Zamorano Cuervo and Grandvaux, 2020). To explore whether Gal-9-promoted virus entry depends on ACE2 binding, we treated cells with an anti-ACE2 antibody that competitively binds to the receptor. As expected, the anti-ACE2 antibody blocked SARS-2-S, but not VSV-G, entry in a dose-dependent manner (*P* < 0.05) (Figure 2C) and also blocked Gal-9-enhanced SARS-2-S infection (*P* < 0.01) (Figure 2D), indicating that Gal-9 facilitates SARS-CoV-2 entry in an ACE2-dependent manner.

**Figure 3:**
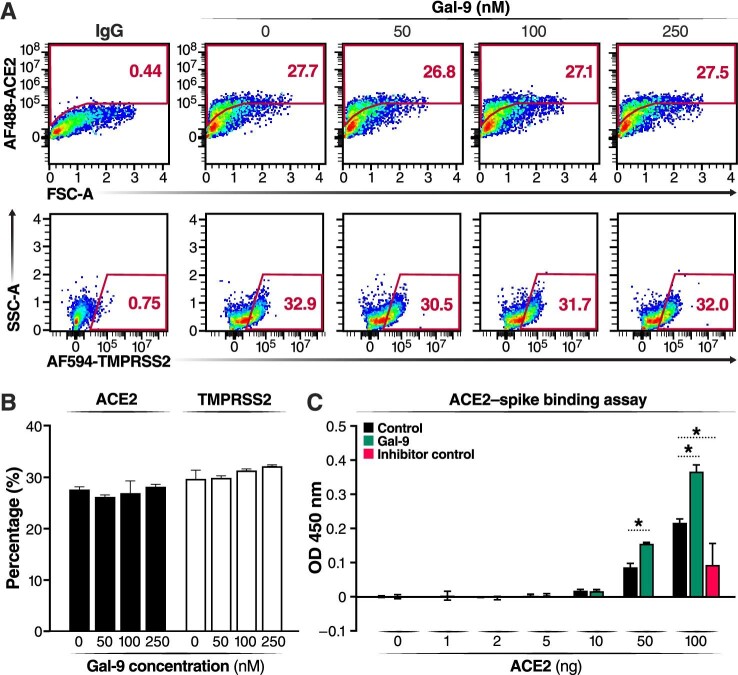
Gal-9 enhances the binding of SARS-CoV-2 spike to ACE2 without affecting ACE2 and TMPRSS2 cell-surface expression. (**A**) Representative plot describing the protein levels of ACE2 and TMPRSS2 on the surface of Calu-3 cells treated with Gal-9. Calu-3 cells were treated with Gal-9 at the indicated concentrations for 24 h. Cells were then washed and detached before antibody staining for flow cytometry. (**B**) Percentage of cells expressing ACE2 or TMPRSS2 on the cell surface was determined by flow cytometry. (**C**) The dose-course of SARS-CoV-2 spike–ACE2 binding activity was measured by reading the absorbance at 450 nm. Data are representative of the results of three independent experiments (mean ± SEM). Statistical significance was analyzed by the *t*-test. *P ≤ 0.05.

We then investigated the potential mechanisms underlying the Gal-9-mediated enhancement of SARS-CoV-2 viral entry. SARS-CoV-2 can use the endosomal cysteine proteases cathepsin B and L (CatB/L) and the serine protease TMPRSS2 to prime entry (Jackson et al., 2022). Only TMPRSS2 activity is essential for viral spread and pathogenesis in the infected host, whereas CatB/L activity is dispensable. In Calu-3 cells that express ACE2 and TMPRSS2 (Figure 3A), SARS-CoV-2 entry was demonstrated to be primed by TMPRSS2 (Hoffmann et al., 2020). However, Gal-9 exhibited no effects on ACE2 and TMPRSS2 expression on Calu-3 cell surface (Figure 3B), suggesting that Gal-9 facilitates virus entry independently of ACE2 or TMPRSS2 induction. We also investigated the direct impact of Gal-9 on the interaction between the SARS-CoV-2 spike protein and the ACE2 entry receptor, using purified spike and ACE2 proteins with an established sandwich enzyme-linked immunosorbent assay (ELISA) protocol (Sama et al., 2020). Gal-9 significantly enhanced the binding between ACE2 and spike (*P* < 0.05) (Figure 3C), indicating that Gal-9 facilitates virus entry and viral replication by strengthening ACE2 and spike interaction.

### Gal-9-mediated enhancement of SARS-CoV-2 entry is glycan-dependent

**Figure 4:**
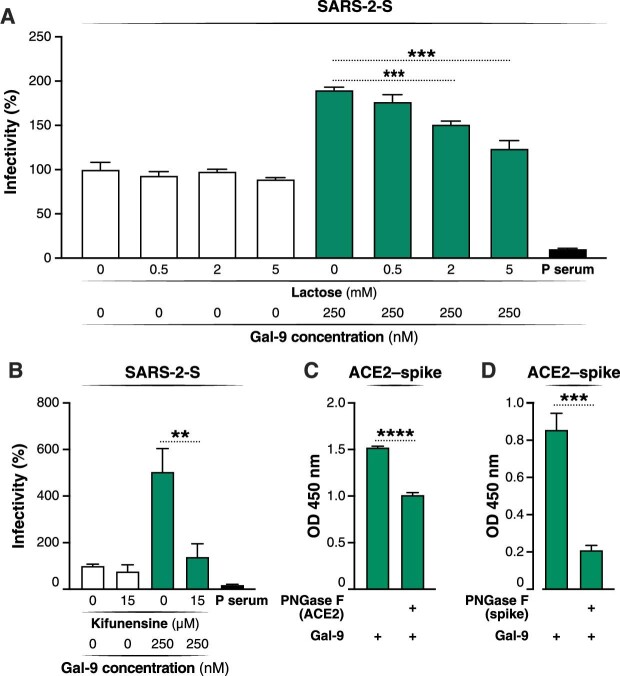
Gal-9-mediated enhancement of SARS-CoV-2 entry is glycan-dependent. (**A** and **B**) The effect of lactose or kifunensine on Gal-9-enhanced SARS-CoV-2 infection was evaluated by measuring luciferase activity at 24 hpi. (**A**) Calu-3 cells were pre-treated with Gal-9 and lactose at the indicated concentrations for 6 h and then inoculated with SARS-2-S pseudotyped virus in solutions containing Gal-9 and lactose at the indicated concentrations. (**B**) Calu-3 cells were pre-incubated with kifunensine for 24 h, followed by treatment with Gal-9 and kifunensine for 6 h, and then inoculated with SARS-2-S pseudotyped virus in solutions containing Gal-9 and kifunensine at the indicated concentrations. Positive serum predetermined to possess anti-SARS-CoV-2 neutralizing activity was used as a positive control. Luciferase signals obtained in the absence of Gal-9 and lactose (**A**) or in the absence of Gal-9 and kifunensine (**B**), respectively, were used for normalization. (**C** and **D**) The effect of PNGase F on Gal-9-enhanced binding between spike and ACE2 was measured by reading the absorbance at 450 nm. ACE2 and spike-coated plates were pre-incubated with PNGase F at 37°C for 16 h. Signals obtained in the absence of Gal-9 and PNGase F were used for normalization. Data are representative of the results of three independent experiments (mean ± SEM). Statistical significance was analyzed by the *t*-test. **P ≤ 0.01; ***P ≤ 0.001; ****P ≤ 0.0001.

To explore the impact of Gal-9–glycan interactions on SARS-CoV-2 infection, we first determined whether Gal-9-enhanced SARS-CoV-2 entry was dependent on CRD activity, by using lactose, a competitive inhibitor of galectin carbohydrate-binding activity (Giovannone et al., 2018). Our data demonstrated that lactose treatment significantly abrogated Gal-9-mediated SARS-CoV-2 entry in a dose-dependent manner (*P* < 0.001) (Figure 4A). We then deglycosylated Calu-3 target cells using kifunensine treatment (Elbein et al., 1990). Kifunensine inhibits mannosidase I enzymatic activity within the cell, preventing hybrid and complex N-linked glycosylation of synthesized proteins. Loss of host complex N-glycans led to significant inhibition of Gal-9-enhanced SARS-CoV-2 entry (*P* < 0.01) (Figure 4B). We next investigated the effect of galectin–glycan interactions on the binding between the viral spike protein and ACE2, using PNGase F exposure to chemically remove N-glycans from purified spike or ACE2 glycoprotein (Huang et al., 2021). PNGase F treatment of spike or ACE2 inhibited the effect of Gal-9 on the binding between these two factors (*P* < 0.001) (Figure 4C and D), indicating that galectin–glycan interactions facilitate viral attachment. Collectively, these data demonstrate that Gal-9 promotes SARS-CoV-2 attachment and entry into host cells in a glycan-dependent manner.

### Gal-9 promotes SARS-CoV-2-associated expression of pro-inflammatory cytokines

**Figure 5:**
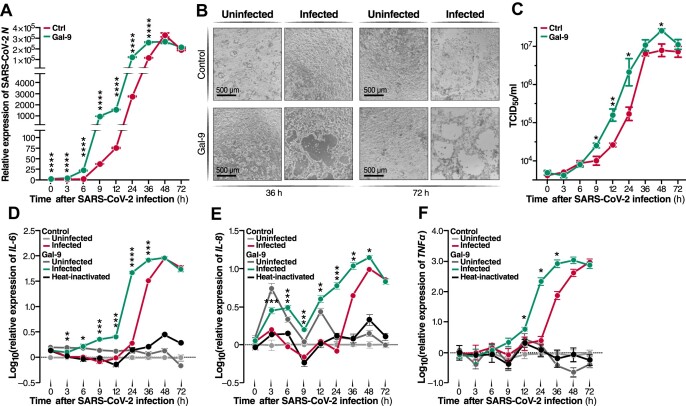
Gal-9 accelerates and increases SARS-CoV-2-mediated inflammatory response. Calu-3 cells were pre-treated with or without Gal-9 (250 nM) for 6 h and then infected with SARS-CoV-2 (MOI = 0.01) in the presence or absence of Gal-9. (**A**) Cells were collected at the indicated times for RNA isolation and RT–qPCR targeting the SARS-CoV-2 *N* gene. (**B**) Calu-3 cells were observed for the development of CPE by bright-field microscopy at 36 and 72 hpi. Scale bar, 500 μM. (**C**) Viral titer (TCID_50_/ml) of supernatant collected at the indicated time points. (**D**–**F**) mRNA levels of pro-inflammatory cytokines *IL-6* (**D**), *IL-8* (**E**), and *TNFα* (**F**) were detected by RT–qPCR at the indicated time points. ‘Heat-inactivated’ indicates cells infected with heat-inactivated (non-infectious) SARS-CoV-2 (negative control). Data are representative of the results of three independent experiments (mean ± SEM). Statistical significance was analyzed by the *t*-test. *P ≤ 0.05; **P ≤ 0.01; ***P ≤ 0.001; ****P ≤ 0.0001.

We next evaluated the temporal characteristics of Gal-9-mediated enhancement of SARS-CoV-2 replication. Our growth curves are compatible with the concept that Gal-9 facilitates SARS-CoV-2 entry, as the expression of the viral *N* gene was significantly increased within 1–3 h post-infection (hpi) in Calu-3 cells compared to untreated controls (*P* < 0.0001) (Figure 5A). Maximum viral yields were detected at 36 hpi with Gal-9 treatment and 48 hpi without Gal-9 treatment, respectively. In accordance with the viral growth kinetic data, microscopy images showed that virus-mediated cytopathic effects (CPE) were much more pronounced in infected cultures treated with Gal-9 at both 36 and 72 hpi than in infected, but untreated cultures (Figure 5B). The enhancement of virus production by Gal-9 over time was also evaluated by TCID_50_ in the supernatant at various time points (Figure 5C). Enhanced release of infectious virus in the presence of Gal-9 was observed as early as 9 hpi (*P* < 0.05), reflecting the effect of Gal-9 on the early stages of the viral life cycle. Collectively, these results reinforce the concept that Gal-9 promotes SARS-CoV-2 viral production through enhancing virus entry.

The host inflammatory response drives much of the pathology, morbidity, and mortality associated with SARS-CoV-2 infection (Merad and Martin, 2020), and Gal-9 was positively correlated with pro-inflammatory mediators and disease severity in COVID-19 patients. Therefore, we sought to determine whether Gal-9 promotes pro-inflammatory cytokine expression, either directly or indirectly, via enhancing viral replication. Uninfected and/or untreated cells were also characterized as a negative control and reference. In the absence of SARS-CoV-2 infection, Gal-9 treatment alone induced *IL-6, IL-8*, and *TNFα* (all *P* < 0.05) gene expression in Calu-3 cells, albeit at modest levels (Figure 5D–F). In the absence of Gal-9, SARS-CoV-2 infection alone potently induced *IL-6, IL-8*, and *TNFα* (all *P* < 0.05) gene expression, starting at 24, 36, and 24 hpi, respectively (Figure 5D–F). In the presence of Gal-9, SARS-CoV-2 infection significantly induced *IL-6, IL-8*, and *TNFα* (all *P* < 0.05) gene expression, starting at 9, 12, and 9 hpi, respectively (Figure 5D–F). Gal-9 treatment of infected cultures potentiated *IL-6* (*P *< 0.001), *IL-8* (*P *< 0.05), and *TNFα* (*P* < 0.05) gene expression compared to SARS-CoV-2 infection alone and also significantly increased *IL-6, IL-8*, and *TNFα* (all *P* < 0.05) gene expression compared to Gal-9 treatment in the absence of SARS-CoV-2 infection (Figure 5D–F). These observations were confirmed, in part, at the protein level by applying a bead-based immunoassay to culture supernatants. Protein measurements confirmed enhanced IL-6 (*P* < 0.05) and IL-8 secretion (*P* < 0.01) by Gal-9 in the presence of SARS-CoV-2 infection (Supplementary Figure S3A and B). TNFα and IL-17A secretion were additionally induced by Gal-9 in infected cells, although not quite achieving statistical significance (Supplementary Figure S3C and D). Considering that the effects of Gal-9 on cytokine expression were relatively negligible in the absence of infection, our data indicate that Gal-9 and SARS-CoV-2 synergistically promote the expression of pro-inflammatory cytokines in AECs. A formal quantitative analysis of synergy indeed revealed a synergistic impact of combined Gal-9 exposure and SARS-CoV-2 infection on *IL-6* and *TNFα* expression in AECs (Supplementary Figure S4).

### RNA-seq analysis reveals synergistic effects of Gal-9 and SARS-CoV-2 on the host transcriptome

**Figure 6:**
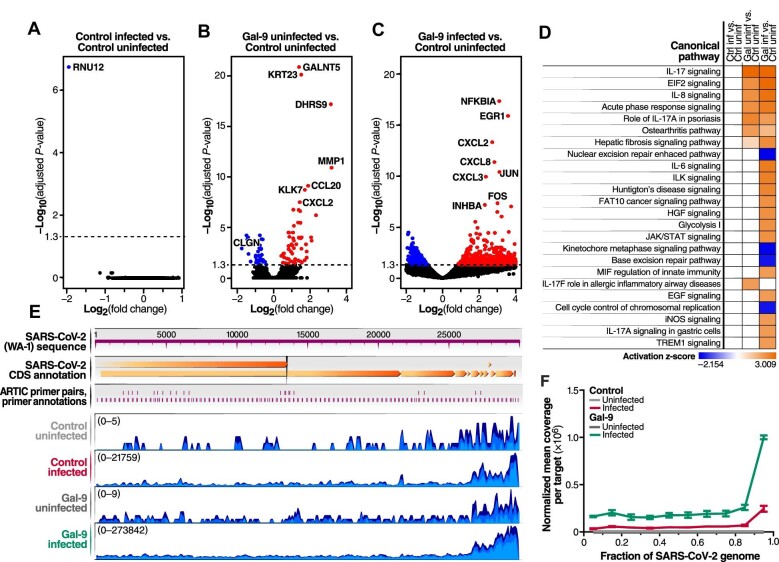
Impact of Gal-9 treatment on the transcriptome of SARS-CoV-2-infected Calu-3 cells. (**A**–**C**) Volcano plots showing the proportion of DEGs in the setting of SARS-CoV-2 infection (Control infected vs. Control uninfected) (**A**), Gal-9 treatment (Gal-9 uninfected vs. Control uninfected) (**B**), and SARS-CoV-2 infection in the presence of Gal-9 (Gal-9 infected vs. Control uninfected) (**C**). DEGs (FDR < 0.05) with log2(fold change) > 0 are indicated in red. DEGs (FDR < 0.05) with log2(fold change) < 0 are indicated in blue. Non-significant DEGs are indicated in black. (**D**) Top enriched canonical pathways identified using IPA. The orange- and blue-colored bars in the bar chart indicate predicted pathway activation and inhibition, respectively, based on the z-score. Ctrl, control; Gal, Gal-9; inf, infected with SARS-CoV-2 (MOI = 0.01); uninf, uninfected with SARS-CoV-2. (**E**) Sample coverage tracks from the QIAGEN genome browser depicting SARS-CoV-2 assembly. Sample coverage tracks were obtained by mapping raw sequencing reads to the USA-WA1/2020 reference genome. Mapped read counts of Control uninfected, Control infected, Gal-9 uninfected, and Gal-9 infected groups are 0–5, 0–21759, 0–9, and 0–273842, respectively. (**F**) Normalized mean coverage per targeted region of the SARS-CoV-2 genome. The X-axis represents the relative target region positions of the SARS-CoV-2 genome. Data were normalized to the total read depth for each sample.

To understand the transcriptional impact of Gal-9-mediated enhancement of SARS-CoV-2 infection and immunopathology, we performed RNA-seq analysis on Calu-3 cells infected for 24 h with SARS-CoV-2 in the presence or absence of 250 nM Gal-9. Uninfected and/or untreated cells were also characterized as a negative control and reference for the other three experimental conditions. Differentially expressed gene (DEG) analysis was performed using a false discovery rate (FDR) cutoff of 0.05. Only one protein-coding gene (*RNU12*) was significantly modulated by SARS-CoV-2 infection alone (FDR = 1.89 × 10^−7^) (Figure 6A), while 87 genes were significantly modulated by Gal-9 treatment alone (Figure 6B), including 30 down-regulated and 57 up-regulated genes. SARS-Cov-2 infection in the presence of Gal-9 exhibited a dramatic impact on the host transcriptome, with 1094 DEGs identified (Figure 6C), including 323 down-regulated and 771 up-regulated genes. Using ingenuity pathway analysis (IPA), we analyzed the enriched canonical pathways that overlapped with the DEGs (Figure 6D). Key pro-inflammatory programs, including IL-17, EIF2, IL-6, IL-8, and JAK/STAT signaling pathways, were activated in infected cells in the presence of Gal-9. A detailed interactome depicting Gal-9 and SARS-CoV-2 effects on IL-17 and IL-6 signaling pathway members is shown in Supplementary Figures S5 and S6.

DEGs were also categorized with respect to diseases and host functions, which are listed in Supplementary Figure S7A and B, demonstrating that the inflammatory response, infectious, inflammatory, and immunological disease pathways were activated in the setting of Gal-9 treatment. Interestingly, pathways related to respiratory disease and RNA post-transcriptional modification were specifically activated in cells infected with SARS-CoV-2 in the presence of Gal-9.

We next leveraged the RNA-seq data to examine the impact of Gal-9 treatment on SARS-CoV-2 expression, by aligning sequencing reads against the USA-WA1/2020 reference genome. The number of reads mapping to each region of the viral genome was calculated and interpreted to infer viral expression patterns (Figure 6E). The transcription of SARS-CoV-2 RNA exhibited an uneven pattern of expression along the genome, typically with a minimum depth in the first coding regions with open reading frames 1a and 1b and a maximum depth toward the 3′ end. This skewing likely reflects the relative abundance of these sequences due to the nested transcription of SARS-CoV-2 subgenomic RNAs. All viral transcript variants include the terminal 3′ genome segment (Cao et al., 2021). Importantly, in accordance with our reverse transcription quantitative polymerase chain reaction (RT–qPCR) data, Gal-9 treatment increased the expression of SARS-CoV-2 RNA, resulting in ∼10-fold increase in the number of sequencing reads mapping to the USA-WA1/2020 reference (Figure 6E and F), maintaining and even amplifying the observed 3′ skewing of viral transcripts.

### Gal-9 enhances SARS-CoV-2 replication and increases TNFα expression in primary AECs

**Figure 7:**
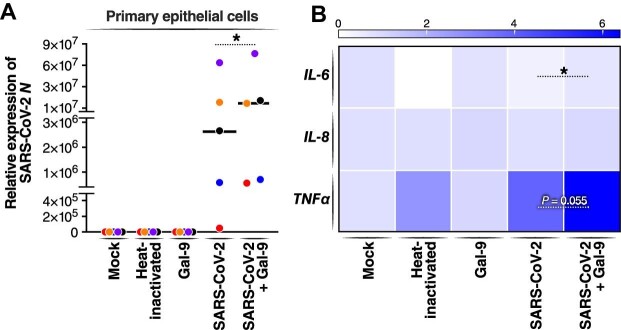
Gal-9 enhances SARS-CoV-2 replication and SARS-CoV-2-induced inflammatory response in human primary AECs. ALI-cultured primary epithelial cells were treated with Gal-9 at a concentration of 250 nM for 6 h and then either infected with SARS-CoV-2 lineage P.1 (MOI = 0.1) or heat-inactivated virus (negative control) or left uninfected (mock control). The infection was carried out by exposing the cells to a solution of Gal-9 (250 nM) in both the apical chamber (250 μl) and the basal compartment (500 μl) of the inserts for a period of 2 h. Cells were washed and supplemented with fresh medium alone or containing Gal-9 (250 nM). (**A**) At 36 hpi, cells were collected for RNA isolation and RT–qPCR analysis of the SARS-CoV-2 *N* gene. Each color represents one donor. (**B**) mRNA levels of pro-inflammatory cytokines *IL-6, IL-8*, and *TNFα* in primary epithelial cells from five different donors. Data represent duplicate experiments for each donor (median). Statistical significance was analyzed by paired one-tailed Wilcoxon tests. *P ≤ 0.05.

Finally, we sought to investigate the effects of Gal-9 treatment on SARS-CoV-2 infection and the inflammatory response in primary human AECs cultured at the air–liquid interface (ALI). Recent studies have reported that human AECs represent the primary gateway for SARS-CoV-2 infection on colonization of a new host (Djidrovski et al., 2021). Rapid viral replication in these cells leads to the release of pro-inflammatory cytokines, which causes airway damage and diminishes patient survival. Thus, our ALI-cultured primary airway epithelial system is useful in modeling the *in vivo* effects of Gal-9 on SARS-CoV-2 infection *ex vivo*. We pre-treated primary AECs from five healthy donors with Gal-9 and then infected them with the SARS-CoV-2 Gamma variant (Pango lineage designation P.1) for 36 h. The Gamma variant was selected based on our observations that the Gamma variant infects primary AECs more efficiently than the founder virus lineage (USA-WA1/2020) (data not shown). Productive infection was observed in all five donor cultures based on SARS-CoV-2 *N* gene expression (Figure 7A). Validating our observations in Calu-3 cells, Gal-9 treatment significantly increased SARS-CoV-2 replication (*P *< 0.05) by up to 2.6-fold (Figure 7A). We also assessed pro-inflammatory signatures at 36 hpi by RT–qPCR and found that SARS-CoV-2 significantly induced *TNFα* expression compared to the mock control (*P* < 0.05) (Figure 7B). The mRNA levels of *IL-6* and *IL-8* were not affected by SARS-CoV-2 infection. Gal-9 treatment significantly increased *IL-6* expression level in the presence of SARS-CoV-2 (*P* < 0.05), and the induction of *TNFα* was observed but failed to achieve statistical significance (*P *= 0.055) (Figure 7B). Taken together, our findings in primary AECs validate and extend our previous observations, confirming that Gal-9 promotes SARS-CoV-2 replication and associated pro-inflammatory signaling in the airway epithelium.

## Discussion

The elevation of plasma Gal-9 levels in COVID-19 cases and severe COVID-19 disease has been confirmed in multiple reports (De Biasi et al., 2020; Bozorgmehr et al., 2021). Here, we leveraged a stable form of recombinant Gal-9 as a proxy for endogenously produced Gal-9 and investigated its impact on SARS-CoV-2 replication and host immune signaling. Our data reveal that Gal-9 enhances SARS-CoV-2 replication in AECs, including ALI-cultured human primary AECs, facilitating cellular entry in a galectin–glycan interaction-dependent manner. Our transcriptomic data show that Gal-9 accelerates and exacerbates several virus-induced pro-inflammatory programs in AECs. These observations are highly relevant to the clinical manifestations and management of COVID-19, suggesting that circulating Gal-9 has a direct impact on viral infectivity and the cytokine milieu in the airway epithelium, which constitutes the critical initial site of SARS-CoV-2 attachment and infection (Mulay et al., 2021). Collectively, our findings complement previous reports highlighting plasma Gal-9 level as a biomarker of severe COVID-19 disease, providing a novel molecular and immunologic framework linking Gal-9 activity to disease pathology (Supplementary Figure S8). Importantly, our data build on a robust literature featuring Gal-9 as a key host factor regulating viral immunopathogenesis.

We observed potent Gal-9-mediated enhancement of SARS-CoV-2 cellular entry, using both pseudoviral constructs and wild-type replication-competent viruses. Previous studies have demonstrated that Gal-9 promotes HIV entry through retaining protein disulfide isomerase on CD4^+^ T cell surface (Bi et al., 2011). Therefore, we initially hypothesized that Gal-9 promoted SARS-CoV-2 entry by retaining or increasing viral receptor expression on the cell surface. However, our flow cytometry data revealed no impact of Gal-9 exposure on the cell-surface expression of ACE2 or TMPRSS2. These data were further validated by our transcriptomic analysis, which failed to show any modulation of ACE2 or TMPRSS2 mRNA transcripts by Gal-9. Studies have indicated roles for Gal-9 in bridging pathogen glycans to host cell surface glycans to promote target cell attachment (Baum et al., 2014). We, therefore, conducted experiments to determine whether this phenomenon underlies Gal-9-mediated enhancement of SARS-CoV-2 entry. Our data confirm the hypothesis that facilitating viral binding and attachment enhances virus entry. This conclusion was supported by three pieces of evidence. (i) The attachment of SARS-CoV-2 virions to the cell surface is enhanced by Gal-9 through galectin–glycan interactions. (ii) Gal-9 directly enhances the binding of the viral spike protein to the ACE2 receptor. (iii) Gal-9 effects on SARS-CoV-2 growth kinetics reflect the facilitation of an immediate-early viral life cycle stage. These findings are compatible with a provocative hypothesis reported by Arciniegas et al. (2021) that multiple galectins act in concert through N- and O-linked glycans on the SARS-CoV-2 spike to form a galectin–glycan lattice on the virion surface, promoting viral attachment and penetration. Lujan et al. (2022) have recently shown that galectins, including Gal-1, Gal-3, and Gal-9, can promote cell-surface binding, internalization, and cell invasion of many sexually transmitted pathogens, including bacteria, parasites, and viruses. This enhancement is mediated by bridging the pathogen surface and receptors on host tissues in a glycosylation-dependent manner (Lujan et al., 2022). Our data further reveal that the cellular entry of VSV-G, like SARS-CoV-2, is enhanced by Gal-9 exposure, suggesting that entry enhancement of multiple viral taxa could be mediated by galectin–glycan lattice interactions.

Accumulating evidence suggests that fatal COVID-19 is characterized by a profound cytokine storm (Fajgenbaum and June, 2021). The overproduction of pro-inflammatory cytokines, such as IL-6, IL-8, TNFα, and IL-1β, leads to an increased risk of vascular hyperpermeability, acute lung injury, multiorgan failure, and eventually death when high cytokine concentrations are unabated over time (Costela-Ruiz et al., 2020). In direct relation to this phenomenon, our transcriptomic analysis revealed that Gal-9 and SARS-CoV-2 infection synergistically induced the expression of key pro-inflammatory programs in AECs, including the IL-8, IL-17, EIF2, and TNFα signaling pathways. These data were validated in part at the protein level using a bead-based immunoassay to characterize the AEC secretome. Bozorgmehr et al. (2021) demonstrated highly significant positive correlations of plasma Gal-9 levels with a wide range of pro-inflammatory biomarkers in COVID-19 patients. They further demonstrated that Gal-9 treatment of monocytes *in vitro* enhanced the expression and production of key pro-inflammatory molecules associated with severe COVID-19 disease (Bozorgmehr et al., 2021). Gal-9 induces the secretion of inflammatory cytokines in several immune cell lineages, including monocyte-derived macrophages and neutrophils (Nobumoto et al., 2009; Querol Cano et al., 2019). Our findings validate and extend these observations to SARS-CoV-2 infection of the airway epithelium.

Importantly, the combined effects of SARS-CoV-2 infection and Gal-9 exposure on pro-inflammatory signaling are much stronger than the effect of either Gal-9 or SARS-CoV-2 alone, reflecting a synergistic interaction. We observed little transcriptional perturbation by SARS-CoV-2 alone after 24 h of infection, which is consistent with other reports that profound transcriptional changes are only evident after 48 h (Blanco-Melo et al., 2020; Subramaniyan et al., 2021). Interestingly, the IL-17 signaling pathway was significantly activated after Gal-9 treatment in the presence or absence of SARS-CoV-2. Our data are in accordance with a previous report demonstrating the induction of IL-17 signaling by Gal-9 treatment *in vivo* in the setting of sepsis (Kadowaki et al., 2013). For MERS-CoV, SARS-CoV, and now SARS-CoV-2, disease severity has been shown to positively correlate with levels of IL-17 and other T helper 17 cell-related pro-inflammatory cytokines (Mahallawi et al., 2018; Yang et al., 2020; Hasan et al., 2021). IL-17 inhibition has been adopted as a common and successful strategy to reduce the injury associated with inflammatory autoimmune diseases (Pacha et al., 2020). Thus, inhibition or neutralization of Gal-9 would not only decrease SARS-CoV-2 replication but also attenuate IL-17 signaling and other damaging pro-inflammatory cascades.

Our study has limitations that must be considered. Firstly, we focused exclusively on the airway epithelium. SARS-CoV-2 is capable of infecting other cell lineages, including monocytes, monocyte-derived macrophages, and microglia (Boumaza et al., 2021; Liu et al., 2021; Jeong et al., 2022). It is possible that Gal-9 does not exert similar effects on viral replication or immune signaling in other target cell types. Secondly, our studies were all performed *in vitro* or in transplant tissue-derived primary epithelial cells *ex vivo*. As Gal-9 exerts conditional, pleiotropic immunomodulatory effects, the net effect of Gal-9 signaling on SARS-CoV-2 pathogenesis cannot be fully appreciated outside of an animal model with a functional immune system. Validation and extension of these *in vitro* and *ex vivo* results in murine, hamster, or non-human primate models of SARS-CoV-2 infection will help evaluating the clinical relevance of our findings. In relevance to the implementation of animal models, our study did not elucidate the principal cell or tissue sources responsible for secreting Gal-9 in the setting of SARS-CoV-2 infection. A study examining the role of Gal-9 in pulmonary fibrosis revealed that immune cells, including alveolar macrophages, lymphocytes, and type II pneumocytes, are the principal sources of Gal-9 production in the diseased lung (Matsumoto et al., 2013). Leveraging an animal model to identify these source compartments will be critical in developing interventions to manipulate Gal-9 signaling as a therapeutic approach.

To the best of our knowledge, the data presented here are the first to show that Gal-9 is directly involved in enhancing SARS-CoV-2 infection and virus-induced pro-inflammatory pathology, exposing an area of vulnerability for further investigation. Our data warrant examination of Gal-9-targeting therapeutic strategies *in vivo*, designed to simultaneously inhibit viral replication and suppress deleterious immune signaling associated with COVID-19 disease. There are existing tools that may be exploited to experimentally manipulate Gal-9 *in vivo*, including administration of exogenous recombinant Gal-9 to enhance signaling (Nishi et al., 2005). Small molecules and promising monoclonal antibodies under investigation in the clinical oncology area (e.g. LYT-200) that antagonize Gal-9 activity may have utility in COVID-19 (Bertino et al., 2019; Filipovic et al., 2021; Yang et al., 2021). On this note, a recent exploratory clinical trial involved administration of a galectin antagonist to individuals with COVID-19 disease and revealed beneficial effects of galectin inhibition on disease outcomes (Sigamani et al., 2020). This represents a provocative and promising early step in the development of galectin-based therapeutics for SARS-CoV-2 infection in the future.

## Materials and methods

### Cell lines

The Calu-3 human lung adenocarcinoma epithelial cell line was obtained from ATCC (ATCCHTB-55) and cultured in Eagle's minimum essential medium (EMEM). Vero E6 cells were purchased from ATCC (CRL-1586) and cultured in Dulbecco's modified Eagle's medium (DMEM). Vero E6 cells stably expressing TMPRSS2 (Vero E6-TMPRSS2) were established and cultured in DMEM in the presence of puromycin (1 µg/ml). All media were supplemented with 10% fetal bovine serum (FBS) and 1% penicillin/streptomycin. All cells had been previously tested for mycoplasma contamination and incubated at 37°C in a humidified atmosphere with 5% CO_2_.

### Primary AECs

Human bronchi were harvested from five explanted healthy lungs. The tissue was submerged and agitated for 1 min in PBS with antibiotics and 5 mM dithiothreitol to wash and remove mucus. After three washes, the tissue was placed in DMEM with 0.1% protease and antibiotics overnight at 4°C. The next day, the solution was agitated, and the remaining tissue was removed. Cells were centrifuged at 300× *g* (4°C) for 5 min, and then the cell pellet was resuspended in 0.05% trypsin-EDTA and incubated for 5 min at 37°C. The trypsinization reaction was neutralized with 10% FBS in DMEM, and then cells were filtered through a cell strainer and centrifuged at 300× *g* (4°C) for 5 min. The cell pellet was resuspended in DMEM with 10% FBS, and 10 μl was stained with trypan-blue and counted on a hemocytometer. Approximately 75000 cells were plated onto each 6 mm/0.4 mm Transwell ALI insert after treatment with the FNC coating mixture (Athena Enzyme Systems, 0407). DMEM and ALI medium with 10% FBS were added in equal volumes to each basal compartment, and cultures were incubated at 37°C with 5% CO_2_. The next day, the medium was removed, and basal compartments were washed with PBS and antibiotics. ALI medium was then added to each basal compartment and changed every 3 days until cells were ready for use on Day 28.

### Viruses

SARS-CoV-2 viruses (isolate USA-WA1/2020 and isolate lineage P.1) were obtained from BEI Resources of the National Institute of Allergy and Infectious Diseases. Viruses were propagated in Vero E6-TMPRSS2 cells in DMEM with 2% FBS and 1% penicillin/streptomycin, and viral stocks were stored at −80°C. Viral titer was measured in Vero E6 cells by the TCID_50_ assay. All the studies involving live viruses were conducted at the Vitalant Research Institute BSL-3 under approved safety protocols.

### Ethics statement

The studies involving human participants were reviewed and approved by the Human Research Protection Program, University of California, San Francisco. The patients/participants provided the written informed consent to participate in this study.

### Cytotoxicity assay

The cytotoxic effect of Gal-9 on Calu-3 cells was determined using an MTT assay kit (Abcam, ab211091) according to the manufacturer's guidelines. In brief, Calu-3 cells cultured in 96-well plates were incubated with different concentrations of Gal-9 (0–5000 nM). After 48 h, the medium was removed, and 100 μl of MTT reagent (1:1 dilution in serum-free DMEM) was added to each well and incubated for 3 h at 37°C. Then, the medium was removed, and 150 μl of MTT solvent was added to each well. Quantification was performed by reading the absorbance at 590 nm wavelength. The data from three independent experiments were used to calculate the CC_50_ by non-linear regression using GraphPad Prism 8.0 software.

### SARS-CoV-2 infection and Gal-9 administration

The stable form of recombinant Gal-9 was used in all experiments (Nishi et al., 2005; Abdel-Mohsen et al., 2016). Calu-3 cells were seeded at 0.5 × 10^6^ cells per well in 0.5 ml volume using a 24-well plate or at 1 × 10^5^ cells per well in 0.1 ml volume using a 96-well plate. The following day, cells were pre-treated with or without Gal-9 for 6 h. Then, viral inoculum (MOI = 0.01; 500 μl/well or 100 μl/well) was prepared using EMEM containing the indicated concentrations of Gal-9 and added to the wells. The inoculated plates were incubated at 37°C with 5% CO_2_. Supernatants were collected and stored at −80°C at 24 hpi. Cells were lysed with TRIzol (Thermo Fisher Scientific, 15596026) for RNA extraction or fixed with methanol:acetone (1:1) for immunofluorescence assays.

For infection of ALI-cultured primary AECs, SARS-CoV-2 (diluted in ALI medium, MOI = 0.1) was added to the apical chamber (250 μl) and the basal compartment (500 μl) of the inserts. Then, the cultures were incubated for 2 h at 37°C (5% CO_2_) to enable virus entry. Subsequently, the cells were washed, and fresh ALI medium (500 μl) was added to the basal compartment. Cells were incubated at 37°C (5% CO_2_) and harvested for analysis at 36 hpi.

### TCID_50_ assay

Viral production by infected cells was measured by quantifying TCID_50_. Vero E6 cells were plated in 96-well plates at 5 × 10^4^ cells per well. The next day, supernatants collected from Calu-3 cells were subjected to 10-fold serial dilutions (10^1^–10^11^) and inoculated onto Vero E6 cells. The cells were incubated at 37°C with 5% CO_2_. Three to five days post-infection, each inoculated well was evaluated for the presence or absence of viral CPE. TCID_50_ was calculated based on the method of Reed and Muench (1938).

### Immunofluorescence microscopy

Cells were washed with 1× PBS and then fixed and permeabilized with cold methanol:acetone (1:1) for 10 min at 4°C. Next, cells were washed with 1× PBS and incubated in blocking buffer (5% goat serum, Seracare Life Sciences Inc., 55600007) at room temperature for 30 min. Cells were then incubated with a primary antibody (monoclonal rabbit anti-SARS-CoV-2 N antibody, GeneTex, GTX135357) in 1× PBS (1:1000) overnight at 4°C. The following day, cells were washed three times with 1× PBS and incubated with a secondary antibody [goat anti-rabbit IgG (H + L), FITC (Thermo Fisher, 65–6111)] in 1× PBS (1:200) for 1 h at 37°C. Then, cells were washed three times with 1× PBS and incubated with DAPI (300 nM, Thermo Fisher Scientific, D1306) for 5 min at room temperature. Images were acquired using a fluorescence microscope.

### RNA-seq and IPA

RNA concentration and quality were measured using High Sensitivity RNA ScreenTape Analysis (Agilent, 5067-1500). cDNA libraries were prepared using the Illumina TruSeq Stranded Total RNA Library Prep Kit (Illumina, 20020597), and sequencing was performed on the Illumina Nextseq 550 Platform, generating 75 bp paired-end reads. The quality of raw sequencing reads was assessed using FastQC. DEGs were identified by GSA or ANOVA in Partek® Flow® imported into the QIAGEN IPA software application. IPA was used to identify gene ontologies, pathways, and regulatory networks to which DEGs belonged, as well as upstream regulators (Krämer et al., 2014). Reads were also aligned to the SARS-CoV-2 isolate WA-1 and analyzed using the QIAGEN CLC Genomics Workbench.

### Pseudovirus entry assay

Calu-3 cells were plated into 96-well plates. The following day, cells were pre-treated with the indicated concentrations of Gal-9 for 6 h. In order to block ACE2 on the cell surface, cells were pre-treated with the indicated concentrations of an anti-ACE2 antibody (R&D Systems, AF933) for 1 h. An unrelated anti-goat IgG antibody (R&D Systems, AB-108-C) was used as a control. Pseudovirus harboring either SARS-CoV-2 spike or VSV-G glycoprotein was diluted in EMEM containing the indicated concentrations of Gal-9 and then added to Calu-3 cells. Controls included wells with serum predetermined to possess neutralizing activity. Cells were incubated for 24 h at 37°C with 5% CO_2_. Supernatants were then removed, cells were lysed, and luciferase activity was read using a commercial substrate (Promega, E1500).

### Flow cytometry

Cells were detached with 10% EDTA containing Zombie NIR (1:300, BioLegend, 423105) for 10 min at 37°C. Then, cells were washed three times with 1× PBS and incubated with human ACE2 Alexa-Fluor 488-conjugated antibody (R&D Systems, FAB9332G), human TMPRSS2 Alexa-Fluor 594-conjugated antibody (R&D Systems, FAB107231T), or human Gal-9 PE-conjugated antibody (Biolegend, 348905) for 30 min at room temperature. Cells were washed three times with 1× PBS again. Analytical flow cytometry was performed with a BD LSRII flow cytometer. Data were analyzed using FlowJo.

### ELISA-based assessment of SARS-CoV-2 spike binding to ACE2

Assessment of Gal-9 effect on the binding of the SARS-CoV-2 spike to human ACE2 was performed using a commercially available spike–ACE2 binding assay kit (CoviDrop SARS-CoV-2 spike–ACE2 Binding Activity/Inhibition Assay Kit, EPIGENTEK, D-1005-48) following the protocol provided by the manufacturer. Gal-9 or the positive inhibitor control was mixed with the indicated amounts of recombinant human ACE2 protein, and then added to an ELISA plate coated with recombinant SARS-CoV-2 spike protein and incubated at 37°C for 60 min. Unbound ACE2 was removed. The amount of captured ACE2, which is proportional to ACE2 binding activity, is then recognized by an ACE2 detection antibody and measured by reading the absorbance at a wavelength of 450 nm.

### Image analysis

To measure the frequency of infected cells, randomly selected areas were imaged. Each treatment had three replicates. The percentage of GFP^+^ cells was determined by dividing the number of GFP^+^ cells by the number of DAPI^+^ cells. All samples were analyzed at the same threshold values. For the quantification of GFP^+^ cells, CellProfiler-3 software was used to determine the fraction of GFP^+^ cells. Briefly, we used the software pipeline CorrectIlluminationCalculate to calculate an illumination correction function for each of the two channels (DAPI, blue; GFP, green). We then used another pipeline, CorrectIlluminationApply, to load each image and correct its illumination using the pre-calculated functions. Next, we ran ColorToGray to change all slides to gray and ran IdentifyPrimaryObjects to identify the number of each channel. Finally, data were exported by using the ExportToSpreadsheet pipeline. The same threshold value was applied to the images of each area.

### Quantitative analysis of synergy

The combined effects of Gal-9 treatment and SARS-CoV-2 infection on pro-inflammatory cytokine expression were analyzed using the SynergyFinder web application, implementing the Bliss Independence model. The Bliss model generates synergy scores from a response matrix (Ianevski et al., 2020).

### Statistical analysis

Statistical analysis was performed using GraphPad Prism version 8 software. Data were presented as mean ± SEM or median. Data were analyzed for statistical significance using an unpaired or paired Student's *t*-test to compare two groups or a paired one-tailed Wilcoxon test. Only *P*-values of 0.05 or lower were considered statistically significant (*P ≤ 0.05; **P ≤ 0.01; ***P ≤ 0.001; ****P ≤ 0.0001).

## Supplementary Material

mjad030_Supplemental_File

## Data availability

The datasets generated for this study are available on request from the corresponding author.

## Notes

**Author contributions:** L.D. and S.K.P. initiated the project and designed the experiments. L.D. and M.S.B. performed the MTT assay. L.D. and A.G. performed and processed RNA-seq. F.D. and J.R.G. isolated and prepared ALI-cultured primary AECs. J.-N.B. analyzed RNA-seq data. L.D., S.T.Y., and L.C.N. quantified GFP^+^ cells. L.D. and P.D. performed multiplex cytokine expression measurements. J.J. and G.S. prepared SARS-CoV-2 stock. T.K. provided human recombinant Gal-9. L.D. and S.K.P. prepared the manuscript. Z.Y.D., J.R.G., and S.K.P. jointly supervised the work.

## References

[bib1] Abdel-Mohsen M. , Chavez L., Tandon R. et al. (2016). Human galectin-9 is a potent mediator of HIV transcription and reactivation. PLoS Pathog. 12, e1005677. 27253379 10.1371/journal.ppat.1005677 PMC4890776

[bib2] Anderson A.C. , Anderson D.E., Bregoli L. et al. (2007). Promotion of tissue inflammation by the immune receptor Tim-3 expressed on innate immune cells. Science 318, 1141–1143. 18006747 10.1126/science.1148536

[bib3] Arciniegas E. , Carrillo L.M., Salgado A. (2021). Potential role of galectin–glycan lattices in SARS-CoV-2 infection and pathogenesis: a hypothesis. Explor. Res. Hypothes. Med. 6, 142–145.

[bib4] Bai G. , Furushima D., Niki T. et al. (2021). High levels of the cleaved form of galectin-9 and osteopontin in the plasma are associated with inflammatory markers that reflect the severity of COVID-19 pneumonia. Int. J. Mol. Sci. 22, 4978. 34067072 10.3390/ijms22094978 PMC8125627

[bib5] Baum L.G. , Garner O.B., Schaefer K. et al. (2014). Microbe–host interactions are positively and negatively regulated by galectin–glycan interactions. Front. Immunol. 5, 284. 24995007 10.3389/fimmu.2014.00284 PMC4061488

[bib6] Bertino P. , Premeaux T.A., Fujita T. et al. (2019). Targeting the C-terminus of galectin-9 induces mesothelioma apoptosis and M2 macrophage depletion. Oncoimmunology 8, 1601482. 31413910 10.1080/2162402X.2019.1601482 PMC6682368

[bib7] Bi S. , Hong P.W., Lee B. et al. (2011). Galectin-9 binding to cell surface protein disulfide isomerase regulates the redox environment to enhance T-cell migration and HIV entry. Proc. Natl Acad. Sci. USA 108, 10650–10655. 21670307 10.1073/pnas.1017954108 PMC3127870

[bib8] Blanco-Melo D. , Nilsson-Payant B.E., Liu W.-C. et al. (2020). Imbalanced host response to SARS-CoV-2 drives development of COVID-19. Cell 181, 1036–1045.e9. 32416070 10.1016/j.cell.2020.04.026 PMC7227586

[bib9] Boumaza A. , Gay L., Mezouar S. et al. (2021). Monocytes and macrophages, targets of severe acute respiratory syndrome coronavirus 2: the clue for coronavirus disease 2019 immunoparalysis. J. Infect. Dis. 224, 395–406. 33493287 10.1093/infdis/jiab044 PMC7928817

[bib10] Bozorgmehr N. , Mashhouri S., Perez Rosero E. et al. (2021). Galectin-9, a player in cytokine release syndrome and a surrogate diagnostic biomarker in SARS-CoV-2 infection. mBio 12, e00384–e00321. 33947753 10.1128/mBio.00384-21 PMC8262904

[bib11] Cao C. , Cai Z., Xiao X. et al. (2021). The architecture of the SARS-CoV-2 RNA genome inside virion. Nat. Commun. 12, 3917. 34168138 10.1038/s41467-021-22785-x PMC8225788

[bib12] Chen P.-K. , Lan J.-L., Huang P.-H. et al. (2021). Interleukin-18 is a potential biomarker to discriminate active adult-onset Still's disease from COVID-19. Front. Immunol. 12, 719544. 34367188 10.3389/fimmu.2021.719544 PMC8343229

[bib13] Costela-Ruiz V.J. , Illescas-Montes R., Puerta-Puerta J.M. et al. (2020). SARS-CoV-2 infection: the role of cytokines in COVID-19 disease. Cytokine Growth Factor Rev. 54, 62–75. 32513566 10.1016/j.cytogfr.2020.06.001 PMC7265853

[bib14] De Biasi S. , Meschiari M., Gibellini L. et al. (2020). Marked T cell activation, senescence, exhaustion and skewing towards TH17 in patients with COVID-19 pneumonia. Nat. Commun. 11, 3434. 32632085 10.1038/s41467-020-17292-4 PMC7338513

[bib15] Del Valle D.M. , Kim-Schulze S., Huang H.-H. et al. (2020). An inflammatory cytokine signature predicts COVID-19 severity and survival. Nat. Med. 26, 1636–1643. 32839624 10.1038/s41591-020-1051-9 PMC7869028

[bib16] Djidrovski I. , Georgiou M., Hughes G.L. et al. (2021). SARS-CoV-2 infects an upper airway model derived from induced pluripotent stem cells. Stem Cells 39, 1310–1321. 34152044 10.1002/stem.3422 PMC8441770

[bib17] Dong E. , Du H., Gardner L. (2020). An interactive web-based dashboard to track COVID-19 in real time. Lancet Infect. Dis. 20, 533–534. 32087114 10.1016/S1473-3099(20)30120-1 PMC7159018

[bib18] Elbein A.D. , Tropea J.E., Mitchell M. et al. (1990). Kifunensine, a potent inhibitor of the glycoprotein processing mannosidase I. J. Biol. Chem. 265, 15599–15605. 2144287

[bib19] Fajgenbaum D.C. , June C.H. (2021). Cytokine storm. Reply. N. Engl. J. Med. 384, e59. 10.1056/NEJMc2036236 33882217

[bib20] Filipovic A. , Wainber Z., Wang J. et al. (2021). Phase1/2 study of an anti-galectin-9 antibody, LYT-200, in patients with metastatic solid tumors. J. Immunother. Cancer 9, A512–A512.

[bib21] Giovannone N. , Liang J., Antonopoulos A. et al. (2018). Galectin-9 suppresses B cell receptor signaling and is regulated by I-branching of N-glycans. Nat. Commun. 9, 3287. 30120234 10.1038/s41467-018-05770-9 PMC6098069

[bib22] Han H. , Ma Q., Li C. et al. (2020). Profiling serum cytokines in COVID-19 patients reveals IL-6 and IL-10 are disease severity predictors. Emerg. Microbes Infect. 9, 1123–1130. 32475230 10.1080/22221751.2020.1770129 PMC7473317

[bib23] Hasan M.Z. , Islam S., Matsumoto K. et al. (2021). SARS-CoV-2 infection initiates interleukin-17-enriched transcriptional response in different cells from multiple organs. Sci. Rep. 11, 16814. 34413339 10.1038/s41598-021-96110-3 PMC8376961

[bib24] Hoffmann M. , Kleine-Weber H., Schroeder S. et al. (2020). SARS-CoV-2 cell entry depends on ACE2 and TMPRSS2 and is blocked by a clinically proven protease inhibitor. Cell 181, 271–280.e8. 32142651 10.1016/j.cell.2020.02.052 PMC7102627

[bib25] Hojyo S. , Uchida M., Tanaka K. et al. (2020). How COVID-19 induces cytokine storm with high mortality. Inflamm. Regen. 40, 37. 33014208 10.1186/s41232-020-00146-3 PMC7527296

[bib26] Hu B. , Guo H., Zhou P. et al. (2021). Characteristics of SARS-CoV-2 and COVID-19. Nat. Rev. Microbiol. 19, 141–154. 33024307 10.1038/s41579-020-00459-7 PMC7537588

[bib27] Huang H.-C. , Lai Y.-J., Liao C.-C. et al. (2021). Targeting conserved N-glycosylation blocks SARS-CoV-2 variant infection in vitro. EBioMedicine 74, 103712. 34839261 10.1016/j.ebiom.2021.103712 PMC8613501

[bib28] Ianevski A. , Giri A.K., Aittokallio T. (2020). SynergyFinder 2.0: visual analytics of multi-drug combination synergies. Nucleic Acids Res. 48, W488–W493. 32246720 10.1093/nar/gkaa216 PMC7319457

[bib29] Iwasaki-Hozumi H. , Chagan-Yasutan H., Ashino Y. et al. (2021). Blood levels of galectin-9, an immuno-regulating molecule, reflect the severity for the acute and chronic infectious diseases. Biomolecules 11, 430. 33804076 10.3390/biom11030430 PMC7998537

[bib30] Jackson C.B. , Farzan M., Chen B. et al. (2022). Mechanisms of SARS-CoV-2 entry into cells. Nat. Rev. Mol. Cell. Biol. 23, 3–20. 34611326 10.1038/s41580-021-00418-x PMC8491763

[bib31] Jeong G.U. , Lyu J., Kim K.-D. et al. (2022). SARS-CoV-2 infection of microglia elicits proinflammatory activation and apoptotic cell death. Microbiol. Spectr. 10, e0109122. 35510852 10.1128/spectrum.01091-22 PMC9241873

[bib32] Kadowaki T. , Morishita A., Niki T. et al. (2013). Galectin-9 prolongs the survival of septic mice by expanding tim-3-expressing natural killer T cells and PDCA-1^+^CD11c^+^ macrophages. Crit. Care 17, R284. 24321251 10.1186/cc13147 PMC4056346

[bib33] Kojima R. , Ohno T., Iikura M. et al. (2014). Galectin-9 enhances cytokine secretion, but suppresses survival and degranulation, in human mast cell line. PLoS One 9, e86106. 24465902 10.1371/journal.pone.0086106 PMC3896437

[bib34] Krämer A. , Green J., Pollard J. et al. (2014). Causal analysis approaches in ingenuity pathway analysis. Bioinformatics 30, 523–530. 24336805 10.1093/bioinformatics/btt703 PMC3928520

[bib35] Lindskog C. (2016). The Human Protein Atlas—an important resource for basic and clinical research. Expert Rev. Proteomics 13, 627–629. 27276068 10.1080/14789450.2016.1199280

[bib36] Liu J. , Li Y., Liu Q. et al. (2021). SARS-CoV-2 cell tropism and multiorgan infection. Cell Discov. 7, 17. 33758165 10.1038/s41421-021-00249-2 PMC7987126

[bib37] Lujan A.L. , Croci D.O., Gambarte Tudela J.A. et al. (2018). Glycosylation-dependent galectin–receptor interactions promote Chlamydia trachomatis infection. Proc. Natl Acad. Sci. USA 115, E6000–E6009. 29891717 10.1073/pnas.1802188115 PMC6042088

[bib38] Lujan A.L. , Croci D.O., Rabinovich G.A. et al. (2022). Galectins as potential therapeutic targets in STIs in the female genital tract. Nat. Rev. Urol. 19, 240–252. 35105978 10.1038/s41585-021-00562-1

[bib39] Machala E.A. , Avdic S., Stern L. et al. (2019). Restriction of human cytomegalovirus infection by galectin-9. J. Virol. 93, e01746–e01718. 30487283 10.1128/JVI.01746-18 PMC6340044

[bib40] Mahallawi W.H. , Khabour O.F., Zhang Q. et al. (2018). MERS-CoV infection in humans is associated with a pro-inflammatory Th1 and Th17 cytokine profile. Cytokine 104, 8–13. 29414327 10.1016/j.cyto.2018.01.025 PMC7129230

[bib41] Matsumoto N. , Katoh S., Yanagi S. et al. (2013). A possible role of galectin-9 in the pulmonary fibrosis of patients with interstitial pneumonia. Lung 191, 191–198. 23321864 10.1007/s00408-012-9446-0

[bib42] Merad M. , Martin J.C. (2020). Pathological inflammation in patients with COVID-19: a key role for monocytes and macrophages. Nat. Rev. Immunol. 20, 355–362. 32376901 10.1038/s41577-020-0331-4 PMC7201395

[bib43] Miyanishi N. , Nishi N., Abe H. et al. (2007). Carbohydrate-recognition domains of galectin-9 are involved in intermolecular interaction with galectin-9 itself and other members of the galectin family. Glycobiology 17, 423–432. 17223646 10.1093/glycob/cwm001

[bib44] Moar P. , Tandon R. (2021). Galectin-9 as a biomarker of disease severity. Cell Immunol. 361, 104287. 33494007 10.1016/j.cellimm.2021.104287

[bib45] Mulay A. , Konda B., Garcia G. et al. (2021). SARS-CoV-2 infection of primary human lung epithelium for COVID-19 modeling and drug discovery. Cell Rep. 35, 109055. 33905739 10.1016/j.celrep.2021.109055 PMC8043574

[bib46] Nishi N. , Itoh A., Fujiyama A. et al. (2005). Development of highly stable galectins: truncation of the linker peptide confers protease-resistance on tandem-repeat type galectins. FEBS Lett. 579, 2058–2064. 15811318 10.1016/j.febslet.2005.02.054

[bib47] Nobumoto A. , Oomizu S., Arikawa T. et al. (2009). Galectin-9 expands unique macrophages exhibiting plasmacytoid dendritic cell-like phenotypes that activate NK cells in tumor-bearing mice. Clin. Immunol. 130, 322–330. 18974023 10.1016/j.clim.2008.09.014

[bib48] Pacha O. , Sallman M.A., Evans S.E. (2020). COVID-19: a case for inhibiting IL-17? Nat. Rev. Immunol. 20, 345–346. 32358580 10.1038/s41577-020-0328-z PMC7194244

[bib49] Patel H. , Ashton N.J., Dobson R.J.B. et al. (2021). Proteomic blood profiling in mild, severe and critical COVID-19 patients. Sci. Rep. 11, 6357. 33737684 10.1038/s41598-021-85877-0 PMC7973581

[bib50] Premeaux T.A. , D'Antoni M.L., Abdel-Mohsen M. et al. (2019). Elevated cerebrospinal fluid galectin-9 is associated with central nervous system immune activation and poor cognitive performance in older HIV-infected individuals. J. Neurovirol. 25, 150–161. 30478799 10.1007/s13365-018-0696-3 PMC6506351

[bib51] Querol Cano L. , Tagit O., Dolen Y. et al. (2019). Intracellular galectin-9 controls dendritic cell function by maintaining plasma membrane rigidity. iScience 22, 240–255. 31786520 10.1016/j.isci.2019.11.019 PMC6906692

[bib52] Reed L.J. , Muench H. (1938). A simple method of estimating fifty percent endpoints. Am. J. Epidemiol. 27, 493–497.

[bib53] Sama I.E. , Ravera A., Santema B.T. et al. (2020). Circulating plasma concentrations of angiotensin-converting enzyme 2 in men and women with heart failure and effects of renin–angiotensin–aldosterone inhibitors. Eur. Heart J. 41, 1810–1817. 32388565 10.1093/eurheartj/ehaa373 PMC7239195

[bib54] Sanz M. , Madrid-Elena N., Serrano-Villar S. et al. (2021). Effect of the use of galectin-9 and blockade of the TIM-3 receptor in the latent cellular reservoir of HIV-1. J. Virol. 95, e02214–e02220. 33361434 10.1128/JVI.02214-20 PMC8092815

[bib55] Sehrawat S. , Reddy P.B.J., Rajasagi N. et al. (2010). Galectin-9/TIM-3 interaction regulates virus-specific primary and memory CD8^+^ T cell response. PLoS Pathog. 6, e1000882. 20463811 10.1371/journal.ppat.1000882 PMC2865527

[bib56] Shahbaz S. , Dunsmore G., Koleva P. et al. (2020). Galectin-9 and VISTA expression define terminally exhausted T cells in HIV-1 infection. J. Immunol. 204, 2474–2491. 32205423 10.4049/jimmunol.1901481

[bib57] Sigamani A. , Ruthra M., Sudhishma et al. (2020). Galectin antagonist use in mild cases of SARS-CoV-2; pilot feasibility randomised, open label, controlled trial. medRxiv, 10.1101/2020.12.03.20238840

[bib58] Subramaniyan B. , Larabee J.L., Bodas M. et al. (2021). Characterization of the SARS-CoV-2 host response in primary human airway epithelial cells from aged individuals. Viruses 13, 1603. 34452468 10.3390/v13081603 PMC8402710

[bib59] Yang R. , Sun L., Li C.-F. et al. (2021). Galectin-9 interacts with PD-1 and TIM-3 to regulate T cell death and is a target for cancer immunotherapy. Nat. Commun. 12, 832. 33547304 10.1038/s41467-021-21099-2 PMC7864927

[bib60] Yang X. , Yu Y., Xu J. et al. (2020). Clinical course and outcomes of critically ill patients with SARS-CoV-2 pneumonia in Wuhan, China: a single-centered, retrospective, observational study. Lancet Respir. Med. 8, 475–481. 32105632 10.1016/S2213-2600(20)30079-5 PMC7102538

[bib61] Zamorano Cuervo N. , Grandvaux N. (2020). ACE2: evidence of role as entry receptor for SARS-CoV-2 and implications in comorbidities. eLife 9, e61390. 33164751 10.7554/eLife.61390 PMC7652413

[bib62] Zhuo Y. , Zhang Y.-F., Wu H.-J. et al. (2017). Interaction between Galectin-9/TIM-3 pathway and follicular helper CD4^+^ T cells contributes to viral persistence in chronic hepatitis C. Biomed. Pharmacother. 94, 386–393. 28772217 10.1016/j.biopha.2017.07.134

