# Supplementary material for: Human galectin-9 potently enhances SARS-CoV-2 replication and inflammation in airway epithelial cells
Source: J Mol Cell Biol. 2023 May 1;15(4):mjad030. doi: 10.1093/jmcb/mjad030 (PMC10668544; doi:10.1093/jmcb/mjad030)
Supplement: mjad030_Supplemental_File [file mjad030_supplemental_file.pdf]

Supplementary Material for

Human Galectin-9 Potently Enhances SARS-CoV-2 Replication and Inflammation in Airway Epithelial Cells

Li Du[1,2], Mohamed S. Bouzidi[1,2], Akshay Gala[1,2], Fred Deiter[3,4], Jean-Noël Billaud[5], Stephen T. Yeung[6], Prerna Dabral[1,2], Jing Jin[1,2], Graham Simmons[1,2], Zain Y. Dossani[1,2], Toshiro Niki[7], Lishomwa C. Ndhlovu[6], John R. Greenland[3,4], Satish K. Pillai[1,2,*]

[1]Vitalant Research Institute, San Francisco 94105, CA, USA.

[2]Department of Laboratory Medicine, University of California, San Francisco, CA 94143-0134, USA.

[3]Department of Medicine, University of California, San Francisco, CA 94143-0410, USA.

[4]Veterans Affairs Health Care System, San Francisco, CA 94121, USA.

[5]QIAGEN Digital Insights, Redwood City, CA 94063, USA.

[6]Division of Infectious Diseases, Department of Medicine, Weill Cornell Medicine, New York, NY 10021, USA.

[7]Kagawa University, Kagawa 760-0016, Japan.

*Corresponding author, e-mail: satish.pillai@ucsf.edu

Materials and methods

RT-qPCR

Total RNA was extracted using the chloroform-isopropanol-ethanol method. 500 ng of RNA was reversed transcribed into cDNA in a 20 µl reaction volume using the RevertAid First Strand cDNA Synthesis Kit (Thermo Fisher Scientific, K1622) in accordance with the manufacturer's instructions. RT-qPCR was performed for each sample using Taqman Universal Master mix II, with UNG (Thermo Fisher Scientific, 4440038) or using PowerUp SYBR Green Master Mix (Thermo Fisher Scientific, A25780) on a ViiA7 Real time PCR system. Primers and probes for detection of the *RNaseP* gene and SARS-CoV-2 *N* gene were obtained from IDT (2019-nCoV RUO Kit (Integrated DNA Technologies, 10006713)). The expression level of the *N* gene was determined relative to the endogenous control of the cellular *RNaseP* gene. Primers for detection of *GAPDH*, *IL-6*, *IL-8*, and *TNFα* were:

GAPDH forward: 5’-AGAAGGCTGGGGCTCATTTG-3’;

GAPDH reverse: 5’-AGGGGCCATCCACAGTCTTC-3’;

IL-6 forward: 5’-GGAGACTTGCCTGGTGAAA-3’;

IL-6 reverse: 5’-CTGGCTTGTTCCTCACTACTC-3’;

IL-8 forward: 5’-ATGACTTCCAAGCTGGCCGTGGCT-3’;

IL-8 reverse: 5’-TCTCAGCCCTCTTCAAAAACTTCTC-3’;

TNFα forward: 5’-CCTCTTCTAATCAGCCCTCTG-3’;

TNFα reverse: 5’-GAGGACCTGGGAGTAGATGAG-3’.

Pseudovirus production

Cells were seeded into 15-cm culture dishes and allowed to attach for 12 hours before transfection with 30 µg viral glycoprotein expression plasmid (pCG SARS-CoV-2 spike and pCAGGS VSV-G) per plate. The transfection medium was changed at 18 hours post-transfection. The expression-enhancing reagent valproic acid was added to a final concentration of 3.75 nM, and the cells were incubated for three-four hours at 37°C with 5% CO_2. Then cells were inoculated with VSVΔG-luc virus at a multiplicity of infection of 0.3 for 4 h before the medium was changed. 24 hours post infection, the supernatants were collected and filtered through a 0.45-µm syringe filter.

Multiplex cytokine analysis

Cytokines in the cell culture supernatants were measured using the human cytokine storm 21-plex procartaplex panel human magnetic bead Luminex assay (Thermo Fisher, EPX210-15850-901), following the manufacturer’s instructions. Supernatants were subjected to 2% Triton X-100 solvent-detergent (SD) mix for virus inactivation prior to quantification. Assay standards and inactivated samples were incubated with fluorescent-coated magnetic beads pre-coated with respective capture antibodies in a 96-well black clear-bottom plate. After washing, biotinylated detection antibodies were incubated with cytokine-bound beads for 30 minutes. Finally, streptavidin-PE was added and incubated for another 30 minutes. Measurements were acquired using the Invitrogen Luminex 200 IS Reader. Data analyses were performed using Bio-Plex Manager™ 6.1.1 (Bio-Rad Laboratories, Hercules, CA, USA). Standard curves were generated with a 5-PL (5-parameter logistic) algorithm, reporting values for both MFI and concentration data.

Measurement of Gal-9 secretion

To measure Gal-9 in culture supernatants, Calu-3 cells and primary AECs were cultured in the absence or presence of SARS-CoV-2 for 24 hours or 36 hours, respectively. The culture supernatants were then collected and were centrifuged at 4000 rpm for 5 minutes to remove cells. Gal-9 expression was measured using a commercial ELISA kit (R&D Systems) according to manufacturer's instructions.

Supplementary Figures

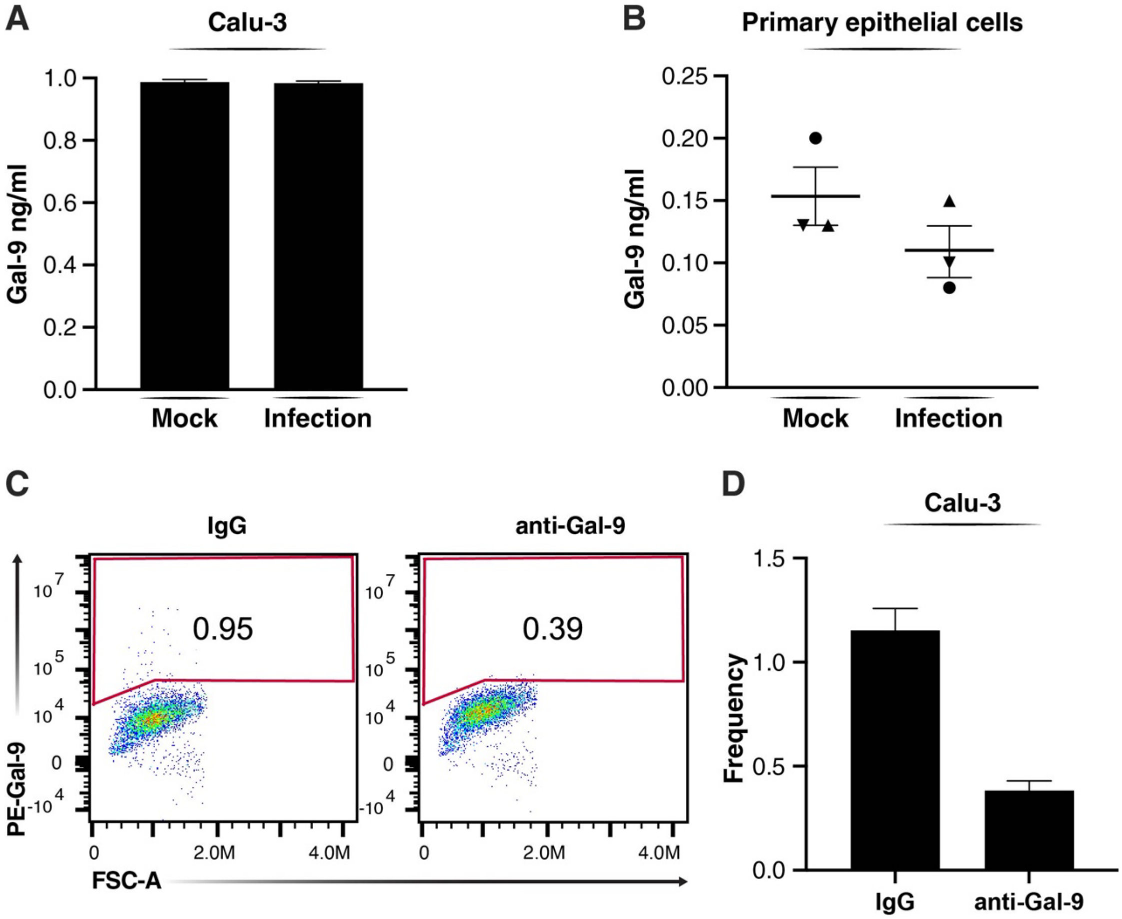

Supplementary Figure S1. Gal-9 expression of human AECs in the absence or presence of SARS-CoV-2. (A-B) ELISA was used to determine the concentration of Gal-9 (ng/ml) in cell culture medium from Calu-3 cells and primary AECs with or without SARS-CoV-2 infection (MOI=0.01 and MOI=0.1, respectively). (C) Representative flow cytometry plot describing the protein levels of Gal-9 on the surface of Calu-3 cells. Cells were washed and detached before antibody staining for flow cytometry. (D) Percentages of cells expressing Gal-9 at the cell surface, measured using flow cytometry. Data are representative of the results of three independent experiments (mean ± SEM) or three different healthy donors (mean ± SEM). Statistical significance was analyzed by *t* test.

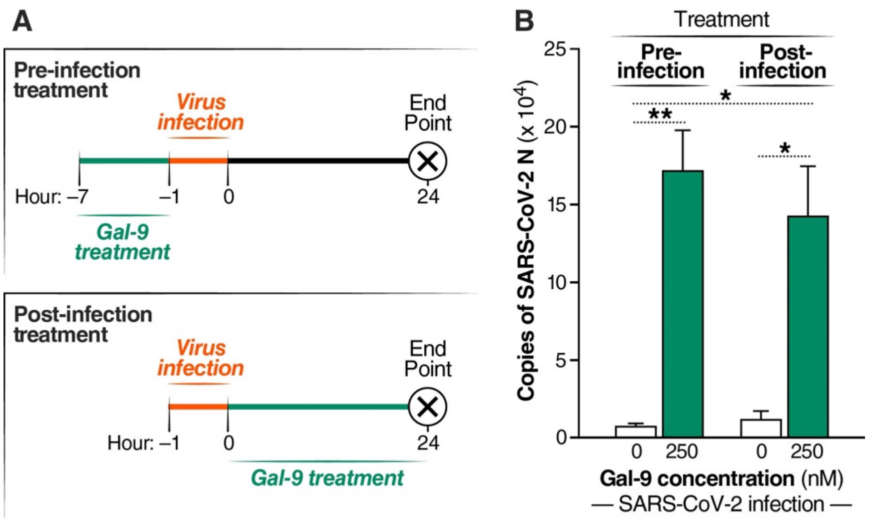

Supplementary Figure S2. Gal-9 promotes SARS-CoV-2 replication during the early stages of the viral life cycle. (A) Schematic timeline of the pre-infection treatment and post-infection treatment experiments. In the pre-infection treatment experiments, Calu-3 cells were pre-treated with 250 nM Gal-9 for six hours. Cells were washed and incubated with SARS-CoV-2 (MOI=0.01) for one hour. Then cells were washed again and were supplemented with fresh media. 24 hpi, cells were harvested for RNA isolation and RT-qPCR targeting the *N* gene. In the post-infection treatment experiments, cells were infected with SARS-CoV-2 (MOI=0.01) for one hour and washed with PBS. Then cells were incubated with 250 nM Gal-9. After 24 hours incubation, cells were harvested for RNA isolation and RT-qPCR targeting the *N* gene. (B) Virus production (measured as viral *N* gene expression) in Calu-3 cells in pre-infection and post-infection treatment scenarios. Copies of SARS-CoV-2 *N* were calculated using the $2^{-\Delta Ct}$ method and *RNaseP* threshold cycle (Ct) values were used for normalization. Data are representative of the results of three independent experiments (mean ± SEM). Statistical significance was analyzed by *t* test. $p \leq 0.05$ [*], $p \leq 0.01$ [**], $p \leq 0.001$ [***], $p \leq 0.0001$ [****].

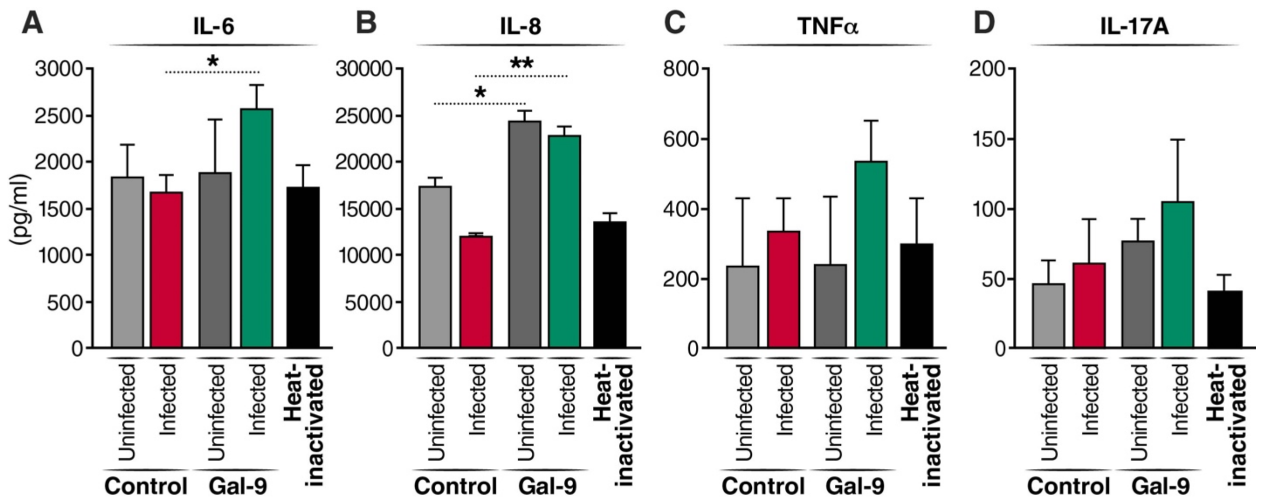

Supplementary Figure S3. Gal-9 treatment and SARS-CoV-2 infection induce secretion of select pro-inflammatory cytokines. (A-D) Supernatants were harvested from Calu-3 cells with indicated treatments at 24 hours post-infection (MOI=0.01). Cytokine protein levels were determined by Luminex assay. Data are representative of the results of three independent experiments (mean ± SEM). Statistical significance was analyzed by *t* test. $p \leq 0.05$ [*], $p \leq 0.01$ [**], $p \leq 0.001$ [***], $p \leq 0.0001$ [****].

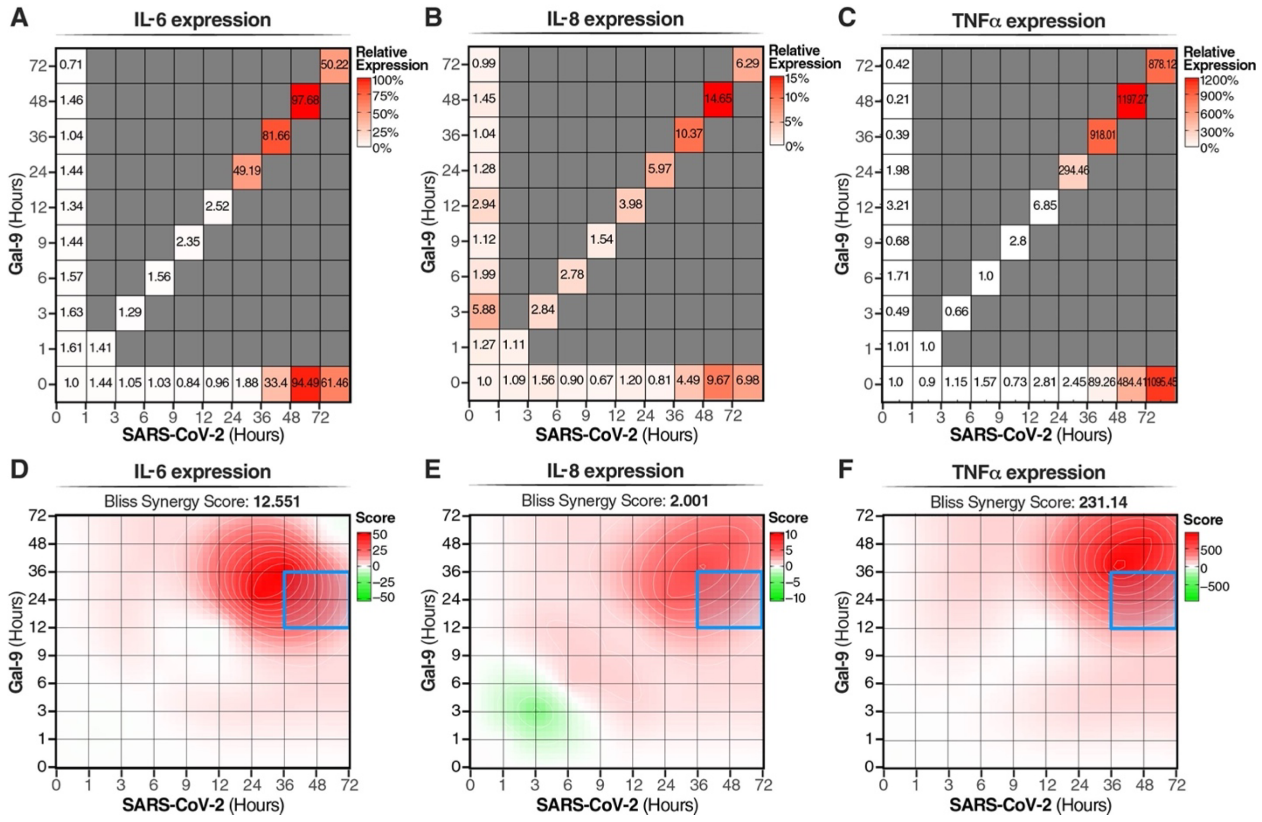

Supplementary Figure S4. Synergistic effect of Gal-9 treatment and SARS-CoV-2 infection on expression of pro-inflammatory cytokines. (A-C) Combinatorial induction of IL-6 (A), IL-8 (B), and TNFα (C) by Gal-9 treatment and SARS-CoV-2 infection (MOI=0.01) are shown by the response matrix of relative expression. (D-F) The synergistic effects on IL-6 (D), IL-8 (E), and TNFα (F) of Gal-9 treatment and SARS-CoV-2 infection are shown by the 2D synergy landscape. When the synergy score is less than -10, the interaction between two treatments is likely to be antagonistic; from -10 to 10, the interaction between two treatments is likely to be additive; larger than 10, the interaction between two treatments is likely to be synergistic.

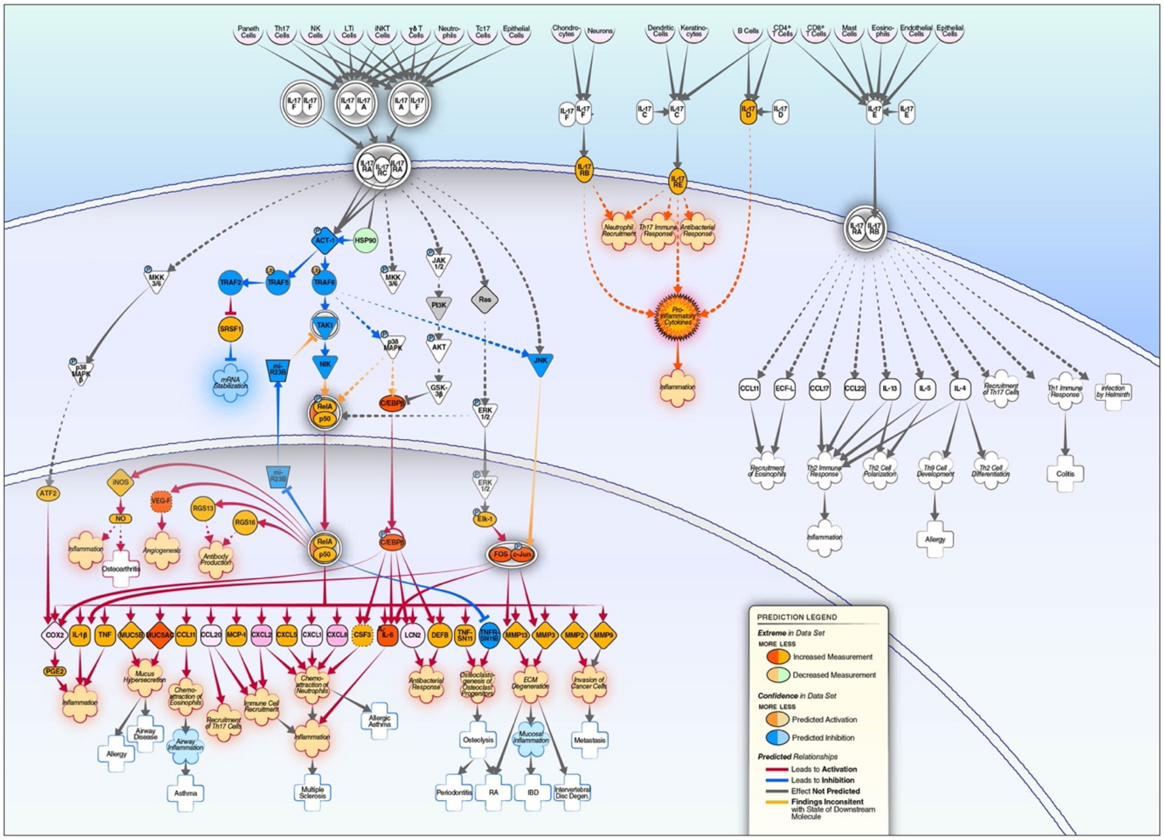

Supplementary Figure S5. Interactome depicting synergistic impact of Gal-9 treatment and SARS-CoV-2 infection on the canonical IL-17 signaling pathway. Predictions were generated by IPA based on the differential expression of genes related to the IL-17 signaling pathway in Gal-9 Infected vs Control Uninfected. Interactome symbols and color codes are defined in the embedded legend.

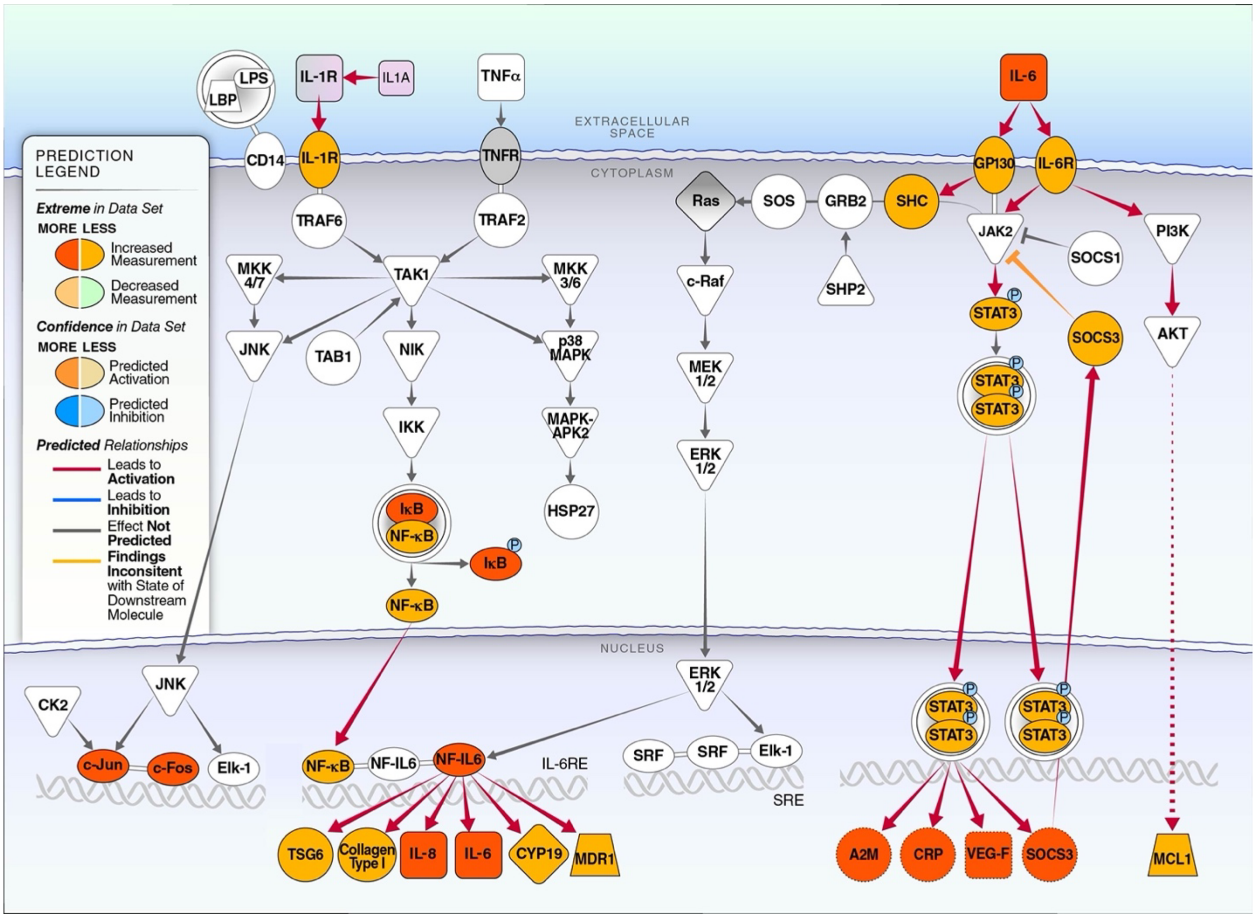

Supplementary Figure S6. Interactome depicting synergistic impact of Gal-9 treatment and SARS-CoV-2 infection on the canonical IL-6 signaling pathway. Predictions were generated by IPA based on the differential expression of genes related to the IL-6 signaling pathway in Gal-9 Infected vs Control Uninfected. Interactome symbols and color codes are defined in the embedded legend.

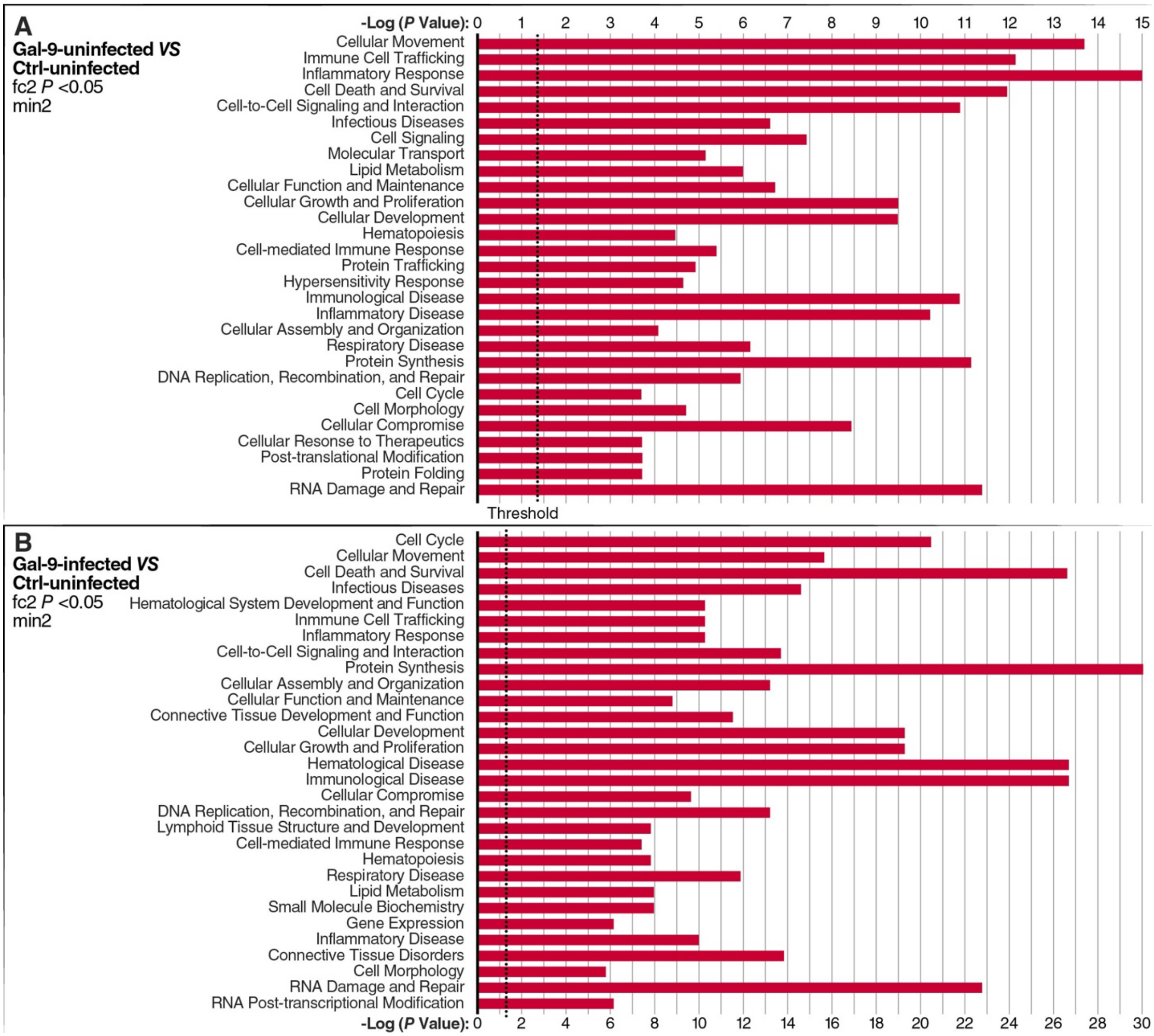

Supplementary Figure S7. Top enriched disease and functional pathways following Gal-9 treatment and SARS-CoV-2 infection. Top enriched diseases and functions (as determined by IPA) in (A) Gal-9 Uninfected vs Control Uninfected and (B) Gal-9 Infected vs Control Uninfected. Diseases and functions were filtered using a FDR<0.05 cutoff with fc>2.

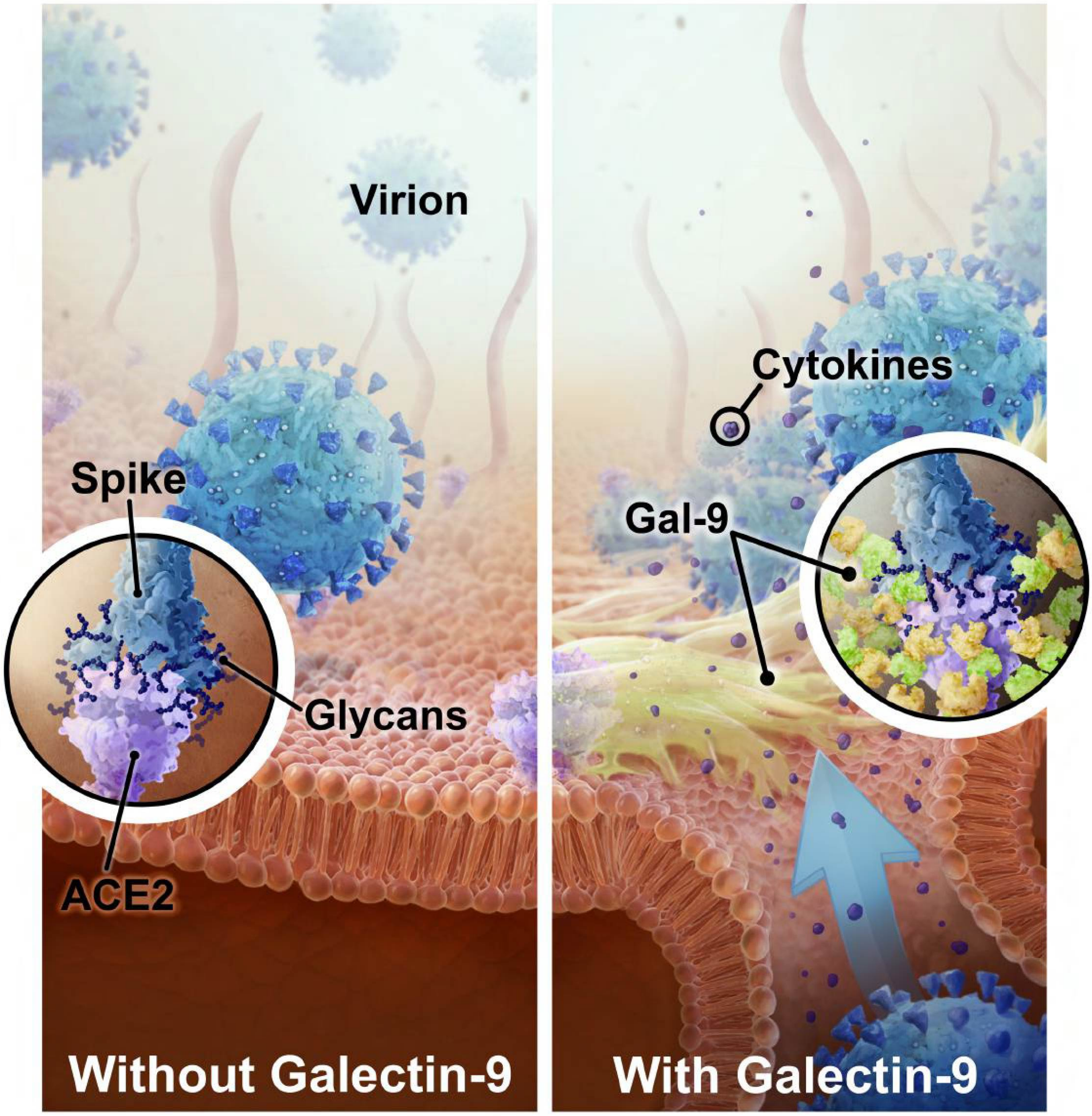

Supplementary Figure S8. Model of how Gal-9 enhances SARS-CoV-2 replication and inflammation in AECs.
